# Function of Armcx3 and Armc10/SVH Genes in the Regulation of Progenitor Proliferation and Neural Differentiation in the Chicken Spinal Cord

**DOI:** 10.3389/fncel.2016.00047

**Published:** 2016-03-03

**Authors:** Serena Mirra, Fausto Ulloa, Irene Gutierrez-Vallejo, Elisa Martì, Eduardo Soriano

**Affiliations:** ^1^Department of Cell Biology, Faculty of Biology, University of BarcelonaBarcelona, Spain; ^2^Centro de Investigación Biomédica en Red Sobre Enfermedades Neurodegenerativas, Instituto de Salud Carlos IIIMadrid, Spain; ^3^Instituto de Biología Molecular de Barcelona, Consejo Superior de Investigaciones Científicas, ParcCientífic de BarcelonaBarcelona, Spain; ^4^Valld'Hebron Institute of ResearchBarcelona, Spain; ^5^Institució Catalana de Recerca i Estudis AvançatsBarcelona, Spain

**Keywords:** Armcx gene cluster, Armc10 gene, progenitor proliferation, mitochondria, neuronal differentiation, spinal cord development

## Abstract

The eutherian X-chromosome specific family of Armcx genes has been described as originating by retrotransposition from Armc10/SVH, a single Arm-containing somatic gene. Armcx3 and Armc10/SVH are characterized by high expression in the central nervous system and they play an important role in the regulation of mitochondrial distribution and transport in neurons. In addition, Armcx/Arm10 genes have several Armadillo repeats in their sequence. In this study we address the potential role of this gene family in neural development by using the chick neural tube as a model. We show that Armc10/SVH is expressed in the chicken spinal cord, and knocking-down Armc10/SVH by sh-RNAi electroporation in spinal cord reduces proliferation of neural precursor cells (NPCs). Moreover, we analyzed the effects of murine Armcx3 and Armc10 overexpression, showing that both proteins regulate progenitor proliferation, while Armcx3 overexpression also specifically controls neural maturation. We show that the phenotypes found following Armcx3 overexpression require its mitochondrial localization, suggesting a novel link between mitochondrial dynamics and regulation of neural development. Furthermore, we found that both Armcx3 and Armc10 may act as inhibitors of Wnt-β-catenin signaling. Our results highlight both common and differential functions of Armcx/Armc10 genes in neural development in the spinal cord.

## Introduction

In early neural development, the neural tube is formed by self-expanding neural precursor cells (NPCs); as neurogenesis proceeds, NPCs located in the ventricular zone progressively exit cell cycle and subsequently give rise to post-mitotic cells accumulating in the mantle zone. Thus, in the development of the spinal cord, a precise, and coordinated regulation of cell cycle progression and differentiation is essential to generate appropriate numbers of neurons. Several signaling pathways, such as Notch, Sonic hedgehog, BMP, and Wnts, are involved in the specification and maintenance of NPCs (Mizutani and Saito, 2005; Cayuso et al., 2006; Han et al., 2008; Nusse et al., 2008; Alvarez-Medina et al., 2009; Bluske et al., 2012; Bowman et al., 2013; Le Dréau et al., 2014). Upon canonical Wnt stimulation, stabilized β-catenin translocates into the nucleus, where it binds to the transcription factors Tcf/LEF (Behrens et al., 1996; Hart et al., 1999), leading to transcriptional activation of multiple target genes such as c-myc and Cyclin D1 and D2 (Megason and McMahon, 2002; MacDonald et al., 2009). The dorsal-ventral gradient of Wnt/β-catenin signaling plays an essential role in maintaining neural progenitor proliferation in the ventricular zone (Chenn and Walsh, 2002; Zechner et al., 2003; Zechner and Bailey, 2004; Kalani et al., 2008). Moreover, Wnt/β-catenin signaling is required for NPC differentiation in neurons (Hirabayashi et al., 2004; Otero et al., 2004; Lie et al., 2005; Lyashenko et al., 2011); more recent studies show that inhibition of the Wnt/β-catenin pathway promotes neuronal differentiation in the intermediate zone of the dorsal neural tube (Xie et al., 2011). Thus, the Wnt/β-catenin signal seems to be essential both to control the size of the progenitor pool and to impinge on the fate of neuronal progenitors, to either proliferate or differentiate.

The Armcx1-6/Armc10 cluster is a recently described gene family encoding for proteins containing multiple Armadillo domains and a mitochondrial targeting signal. In addition to being located in the cell nucleus and the cytosol, Armcx1-6/Armc10 proteins are enriched in mitochondria where they have been shown to regulate mitochondrial neuronal trafficking (López-Doménech et al., 2012). The Armcx genes (Armcx1-6 and the Armcx6-like pseudogene) are clustered on the X chromosome and they are specific to eutherian mammals. This gene cluster originates by retrotransposition from a single Arm-containing gene (Armc10), localized on the 7th chromosome in humans (López-Doménech et al., 2012). Therefore, theArmc10 is the common ancestor gene for the Armcx cluster, and it is present in a single copy in all vertebrates, from Teleosts (López-Doménech et al., 2012).

Several members of Armcx family were initially described as genes lost in a number of human carcinomas (Kurochkin et al., 2001); more recent studies have shown a strong implication of different members of the Armcx/Armc10 family in tumorigenesis (Dall'Era et al., 2007; Jacinto et al., 2007; Zhou et al., 2007; Rohrbeck and Borlak, 2009; Rosales-Reynoso et al., 2010; Iseki H et al., 2012; Montavon et al., 2012; Zeller et al., 2012). Moreover, some members of the Armcx cluster can regulate or are directly regulated by the Wnt signaling pathway (Iseki et al., 2010), which is also implicated in carcinogenesis and tumor progression (Jessen, 2009; McDonald and Silver, 2009; Yao et al., 2011).

To begin to understand the possible role of Armcx/Armc10 family members in neural proliferation and differentiation, we decided to model the expression of these genes in the chick embryo spinal cord, a well-known developmental model. We show that Armcx3 and Armc10 gain-of-function *in vivo* results in negative control of progenitor proliferation. Moreover, Armcx3 overexpression induces neuronal differentiation and the settling of postmitotic neurons in the mantle zone. These functions could be carried out by Wnt/β-catenin regulation, which is inhibited by both Armcx3 and Armc10 proteins. Finally we show that the endogenous Armc10/SVH protein is expressed in chicken spinal cord. Loss-of-function studies confirmed a function of Armc10/SVH in controlling progenitor proliferation and neuronal maturation.

Our data suggest that the function of Armc10/SVH ancestor gene in regulating proliferation/differentiation balance in chicken spinal cord is conserved and further developed in murine Armc10 and Armcx3. Moreover, gene-specific particularities are present in eutherian members of the Armcx/Armc10 family, pointing up both overlapping and differential mechanisms of regulation during neuronal development of eutherian mammals.

## Materials and methods

### Chick embryos

Eggs from white-Leghorn chickens were incubated at 38.5°C in an atmosphere with 70% humidity and staged according to the method of Hamburger and Hamilton (1951). *In ovo* electroporation was performed at stage HH11-12 (48 h of incubation) with DNA plasmids at 3 μg/μl in H_2_O with 50 ng/ml Fast Green, as described previously (Alvarez-Medina et al., 2009). Bromo-deoxyuridine (BrdU, 1 mM; Sigma, St Louis, MO, USA) was injected into the lumen of the chick neural tube at 20 min before harvesting, to label dividing cells. The embryos were recovered at the times indicated (24–48 h post-electroporation, hpe).

### DNA constructs

MouseArmcx3, Armcx3Δ(1-12) and Armc10 sequences were obtained as previously described (López-Doménech et al., 2012; Serrat et al., 2014) and inserted into pCIG (Megason and McMahon, 2002). Full coding Wnt3a, β-catenin-CA, Tcf-VP16 were obtained as previously described (Alvarez-Medina et al., 2009).

To knockdown Armc10 in chick embryos, two short RNA hairpin (shRNA)-based expression vectors were generated (Sh333Fw: 5′-gatcCCCGGGTGGCCTTTCTGTAATT TTCAAGAGAAATTACAGAAAGGCCACCCTTTTTa-3″ and Sh333Rv: 5- agcttAAAAAGGGTGGCCTTTCTGTAATTTCTCTTGAA AATTACAGAAAGGCCACCC GGG-3″; Sh927Fw:5-gatcCCCGCAAGTTGTGAGAATATTATTCAAGAGA TAAT ATTCTCACAACTTGCTTTTTa-3″ and Sh927Rv: 5-agcttAAAAA GCAAGTTGTGAGAATATTA TCTCTTGAA TAAT ATTCTCACAACTTGC GGG-3), cloned into the pSuper vector which contains the pSupershRNA expression cassette and an independent eGFP-encoding cassette and used together (1:1) in the electroporation for Armc10 silencing.

### Evaluation of RNAi efficiency with quantitative real-time-PCR

EGFP-containing plasmid DNAs were electroporated and neural tubes dissected out 24 h later. Single cell suspension was obtained by 10–15 min incubation in Tripsin-EDTA (SIGMA). GFP^+^ cells were sorted by flow cytometry using a MoFlo flow cytometer (DakoCytomation). Total RNA was extracted following the Trizol protocol (Invitrogen). Reverse transcription and real-time PCR were performed according to the manufacturer's instructions (Applied Biosystems) using a PCR quantitative Real-time ABI Prism 7900HT (Applied Biosystems). Oligonucleotides specific for chick Armc10 were designed and used for amplification and normalization (Fw:5′- CAAAGCTCAAGTGCCATCAC-3′; Rv:5′-ATGCCAGCTTCTGAGCAAAT-3, Sigma). Primers specific for chick Gapdh were used for normalization. PCR amplifications were assessed from pools of electroporated neural tube chick embryos (10 embryos/pool), using two independent pools per experimental condition.

### Immunohistochemistry and *in situ* hybridization

Chick embryos were fixed for 2–4 h at 4°C in 4% paraformaldehyde in PB (0.1 M Phosphate Buffer pH 7.2), washed in PBS, and vibratome sectioned (45 μm). For BrdU detection, chick embryos were fixed overnight at 4°C, and the sections were incubated in 2 NHCl for 30 min followed by 0.1 M Na_2_B_4_O_7_ (pH 8.5) rinses, further PBT rinses, and and anti-BrdU incubation. Immunostaining was performed following standard procedures (Lobjois et al., 2008). The following primary antibodies were used: rabbit anti-Armcx3 (1:300, obtained as described in López-Doménech et al., 2012), rabbit anti-GFP (1:500, Invitrogen), rabbit anti-PH3 (1:500, Millipore), rat anti-BrdU (1:500, AbDSerotec), rabbit anti-Armc10 (1:200, Abcam), mouse anti-COXIV (1:500, Invitrogen), mouse anti-Pax7 (1:500, DSHB), mouse anti-Nkx6.1 (1:500 DSHB), mouse anti-Tuj-1 (1:500, Sigma-Aldrich), mouse anti-HuC/D (1:500, Invitrogen), rabbit anti-Sox2 (1:500, Invitrogen), and Mouse Active Caspase-3 (1:300, R&D System). Alexa488-, Alexa562-, and Alexa660-conjugated secondary antibodies were purchased from Invitrogen (Carlsbad, CA). After staining, the sections were mounted in Mowiol, recorded using a Leica SPE confocal microscope, and processed with Adobe Photoshop CS3. Cell counting was carried out on pictures obtained from 4 to 9 different chick embryos per experimental condition. We counted the following groups of cells: GFP+/BrdU+, GFP+/PH3+, GFP+/Sox2+, GFP+/HuC/D+, GFP+/Tuj-1+. In addition, we quantified the percentage of BrdU-positive cells among non-electroporated (GFP-) cells surrounding GFP-positive cells in the following conditions: pCIG, pCIGAlex3, pCIGArmc10, shControl, shArmc10.

For *in situ* hybridization, embryos were fixed overnight at 4°C in 4% PFA in PB, rinsed, and processed for whole-mount RNA *in situ* hybridization following standard procedures (Saade et al., 2013). Chick Armc10 antisense riboprobe was labeled with digoxigenin-D-UTP (Boehringer-Mannheim, Germany) by *in vitro* transcription of a cDNA fragment encoding chick Armc10 [cloneID = ‘ChEST582h22’ adult kidney + adrenal 735pb (nt 318–954); obtained from the chicken EST project (UK-HGMP RC)], using a T3 polymerase (Ambion).

Hybridized embryos were rinsed in PBT, postfixed in 4% PFA, rinsed in PBT, vibratome sectioned, and photographed on a Leica DMR microscope.

### Measurement of HuC/D^+^ or Tuj-1^+^(MZ) areas

The effects of Armcx3, Armcx3Δ(1-12), and Armc10 overexpression or Armc10 silencing on the total number of neurons were assessed by measuring the area occupied by the HuC/D^+^ neurons or the size of the area occupied by Tuj-1^+^ neurons in the MZ. Data were obtained from pictures of coronal sections 48hpe as previously described (Le Dréau et al., 2014). HuC/D^+^ area or Tuj-1^+^ distance were measured using the ImageJ software (National Institutes of Health) for both the control (non-electroporated) and electroporated neural tube sides. The data are presented as the ratios ± s.e.m. obtained by standardizing the values of the electroporated side to the corresponding values of the respective non-electroporated side.

### *In vivo* luciferase-reporter assay

Embryos were electroporated at HH stage 11–12 with the indicated DNA, together with a TOPFLASH luciferase reporter construct containing synthetic TCF-binding sites (Korinek et al., 1998) as well as a Renilla construct (Promega) for normalization. Embryos were harvested after 24 hpe and GFP-positive neural tubes were dissected and homogenized with a douncer in passive lysis buffer (Promega). Firefly- and Renilla-luciferase activities were measured using the Dual Luciferase Reporter Assay System (Promega). Data were obtained from at least two independent experiments (*n* = 6–12 embryos per experimental condition).

### *In silico* analysis

Chick Armc10 protein sequences were obtained by browsing public genome data (www.ensembl.org, www.ncbi.nlm.nih.gov). Domain structure was determined with NCBI, Prosite, InterProScan, and IPSort software.

### Statistical analysis

Quantitative data were expressed as mean ± s.e.m; *n* ≥ 4 embryos per experimental conditions. Data were analyzed using the Student's *t*-test (^*^p < 0.05, ^**^p < 0.01, and ^***^p < 0.001).

## Results

### Forced Armcx3 expression reduces progenitor proliferation

**Figure 1:**
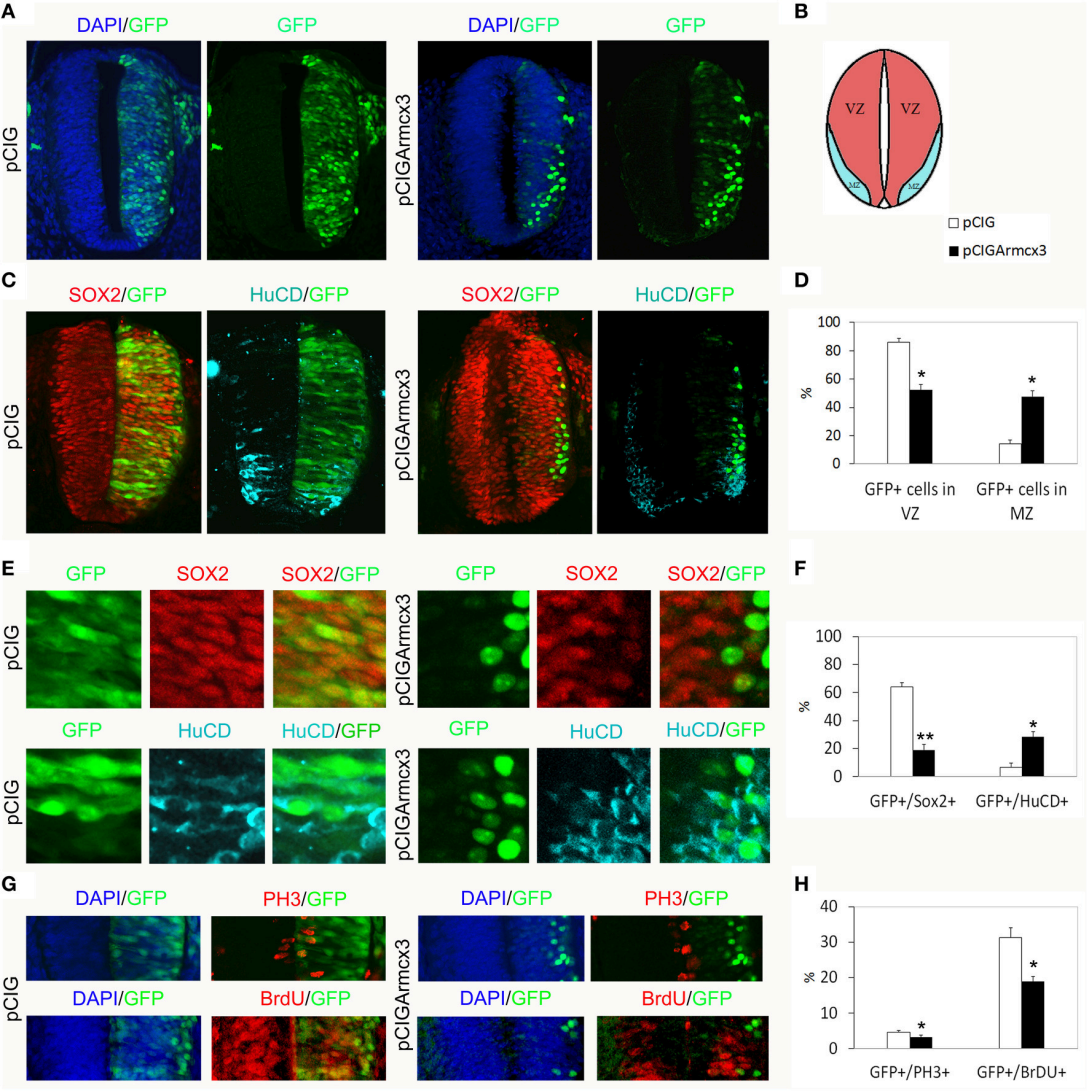
**Armcx3 overexpression reduces progenitor proliferation. (A–D)** Representative transverse sections of neural tubes from embryos electroporated at HH stage 12 with pCIG and pCIGArmcx3 vectors and analyzed at 24hpe with the indicated immunostaining. GFP, Sox2 (red) and HuC/D (blue) stain, respectively, the electroporated cells, the neural progenitors, and the differentiating neurons; Armcx3 overexpressing cells show a lateral distribution from the lumen to the MZ of the neural tube. **(E,F)** Ectopic expression of Armcx3 leads to a dramatic increase in the percentage of HuC/D/GFP-positive cells, concomitant to a decrease in Sox2/GFP-positive cells. **(G,H)** The percentage of GFP-positive electroporated cells positive for PH3 or BrDU decreases in pCIGArmcx3 electroporated embryos. Data represent the mean ± s.e.m. (^*^p < 0.05, ^**^p < 0.01).

In the developing spinal cord, neural progenitors reside in the ventricular zone (VZ). Neural progenitors exit the cell-cycle and migrate to the mantle zone (MZ) where they differentiate in postmitotic neurons. To explore the function of murine Armcx3 in neurogenesis, we electroporated pCIGArmcx3 or the pCIG empty vector as a control into the spinal cord of HH12 chick embryos. Electroporation of the control construct yielded a homogenous distribution of labeled cells through the neural tube, 24hpe (Figures 1A–C). In contrast, expression of pCIGArmcx3 resulted in an altered cellular distribution, with most GFP-positive cells being located at the outer surface of the tube near the MZ (Figures 1A–C). To confirm these findings, we took advantage of Sox2 and HuC/D staining to delineate the VZ and the MZ, respectively (Figure 1C). We confirmed that pCIG-electroporated cells showed an even distribution throughout the tube, whereas Armcx3-expressing cells located in the MZ exhibited a four-fold increase, compared to controls (Figure 1D). To support these findings we quantified the percentage of GFP-positive cells expressing either the progenitor marker Sox2 or the MZ marker HuC/D. Our data show that Armcx3 expression leads to a dramatic increase in the percentage of HuC/D/GFP-positive cells, concomitant with a decrease in Sox2/GFP-positive cells (Figure 1F). These findings suggest that forced Armcx3 expression may induce an early cell cycle exit in progenitor cells.

To better analyze the effect of Armcx3 overexpression on progenitor proliferation, we compared the number of GFP-positive cells co-expressing PH3 (M phase marker) and BrdU (S phase marker) in pCIG and in pCIGArmcx3electroporated embryos; with both markers we found a significant decrease of double-labeled cells in embryos electroporated with Armcx3, suggesting that Armcx3isa negative regulator of cell cycle in the spinal cord *in vivo* (Figures 1G,H).

### Forced Armcx3 expression promotes neural maturation

**Figure 2:**
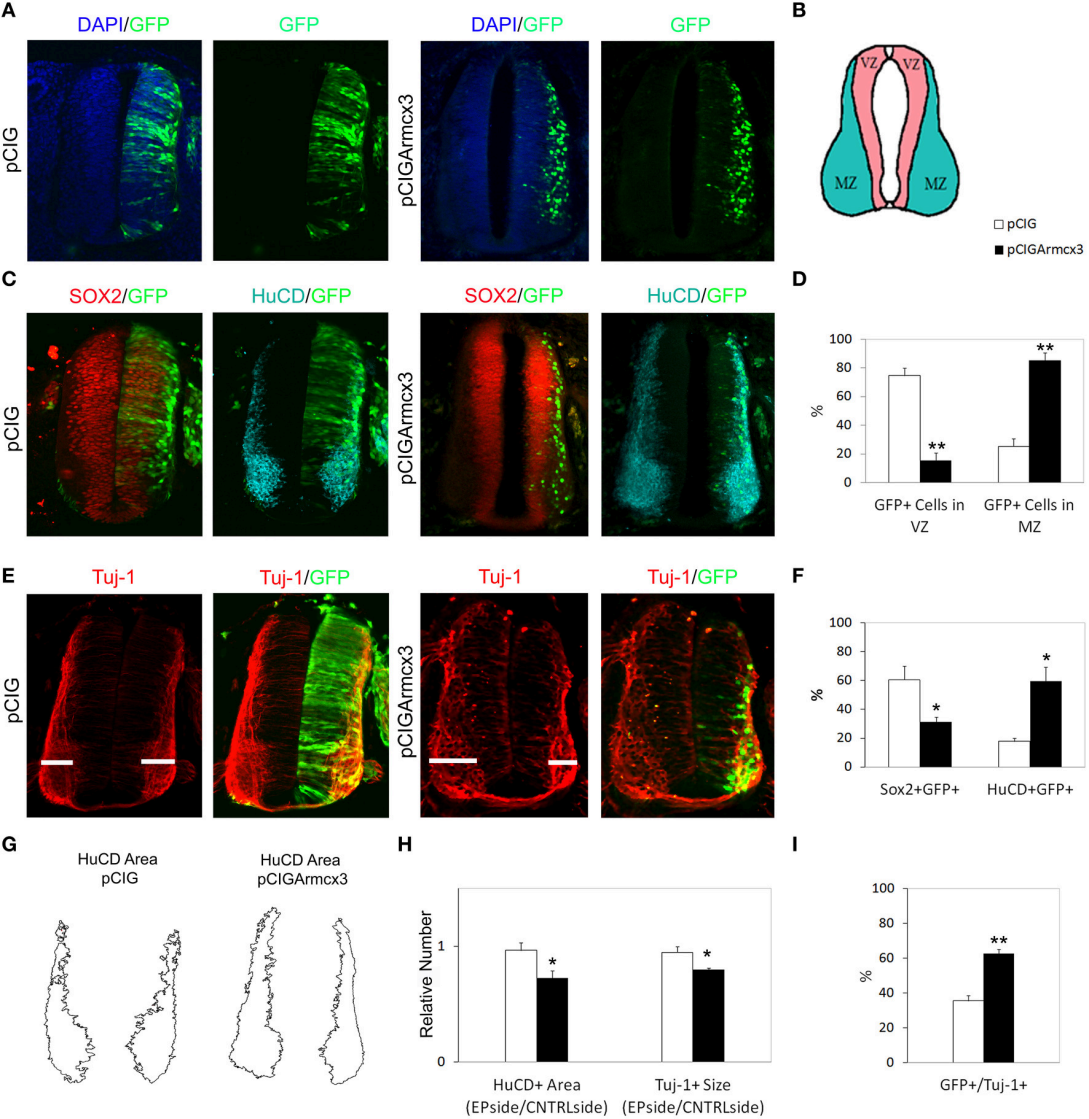
**Armcx3 overexpression promotes neural differentiation. (A–E)** Representative transverse sections of neural tubes from embryos electroporated at HH stage 12 with pCIG and pCIGArmcx3 vectors and analyzed at 48hpe with the indicated immunostaining. Armcx3 overexpressing cells show a lateral distribution from the VZ to the MZ of the neural tube. **(F)** Ectopic expression of Armcx3 leads to an increase in the percentage of HuC/D/GFP-positive cells and a decrease in Sox2/GFP-positive cells (anti-HuC/D, blue; anti-Sox2, red). **(G)** The HuC/D^+^ areas corresponding to the MZ (formed by the differentiating neurons) were defined using ImageJ processing. **(H)** The areas measured for the electroporated side (EP) were standardized to their contralateral controls (CNTRL) and are presented as ratios of the area of MZ (HuC/D^+^ Area); the widths of the Tuj-1-marked region for the electroporated side were standardized to their contralateral controls and are presented as ratios of the size of MZ (Tuj^+^ Size). **(I)** Histogram showing the percentage of electroporated cells (GFP^+^) positive for Tuj-1. Data represent the mean ± s.e.m. (^*^p < 0.05, ^**^p < 0.01).

To analyze the effect of Armcx3 overexpression on neuronal differentiation, we analyzed pCIG and pCIGArmcx3 electroporated embryos 48 h after electroporation (Figure 2A). As with 24hpe embryos, we found that pCIGArmcx3 electroporated embryos exhibited a larger percentage of GFP-positive cells in the MZ (Figures 2A–D). Moreover, we observed a reduction in the proportion of double-labeled GFP/Sox2^+^ cells in pCIGArmcx3-electroporated embryos, concomitant with an increase in the proportion of GFP/HuC/D^+^ cells, compared to controls (Figures 2C,F).

To further strengthen the notion that Armcx3 acts as a promoter of neural differentiation, we analyzed the expression of the neuron-specific marker Tuj-1 in pCIG and pCIGArmcx3 electroporated embryos (Figure 2E). We found that the number of Tuj-1/GFP-positive cells was almost double in Armcx3-electroporated embryos compared to control embryos, thereby confirming that forced Armcx3 expression promotes neuronal maturation (Figure 2I).

To assess the global impact of Armcx3 expression on the size of the differentiating compartment (MZ), we measured the size of Tuj-1^+^and HuC/D^+^ regions. Using both markers we found that Armcx3 expressions leads to smaller compartments occupied by differentiating neurons (Figures 2G,H). Taken together with the above data, the present findings indicate that Armcx3 expression leads to progenitor cell cycle arrest and accelerated neuronal differentiation, thereby resulting in an overall reduction in the number of postmitotic neurons.

### Armcx3 mitochondrial localization is required to control progenitor proliferation and neuronal differentiation

**Figure 3:**
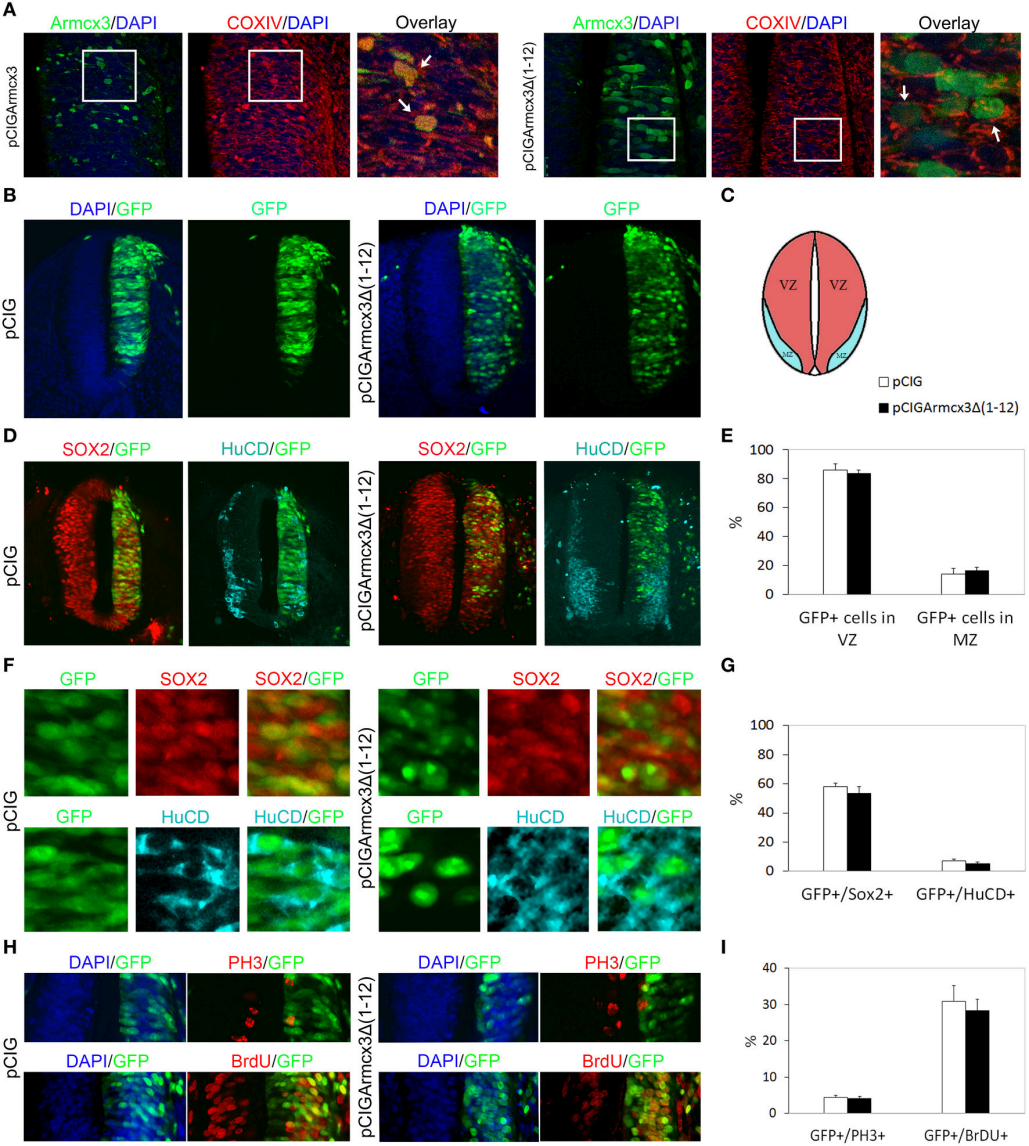
**Armcx3 effects on progenitor proliferation depend on its mitochondrial localization. (A–D,F,H)** Representative transverse sections of chick neural tubes from embryos electroporated at HH stage 12 with the indicated plasmids and processed and analyzed 24hpe as described in Figure 1. Overexpressed Arcmx3 is localized at the mitochondria of neural progenitors as indicated by its colocalization with the mitochondrial marker CoxIV. By contrast, a truncated form of Armcx3 which lacks its mitochondrial targeting sequence (Armcx3Δ1-12) displays a cytoplasmic non-mitochondrial localization **(A)**. Arrows in **(A)** label mitochondria co-localizing with Armcx3 (left panels) but not with the Armcx3Δ(1-12) construct lacking the mitochondrial targeting sequence (right panels). The overexpression of this truncated form neither induced a lateral distribution of progenitors **(B,E)** nor modified the percentage of Sox2/GFP-positive or HuCD/GFP-positive cells **(D–G)**. Consistently, the non-mitochondrial Armcx3 form did not induce changes in the percentage of GFP/PH3 or GFP/BrdU positive cells **(I)**. Data represent the mean ± s.e.m.

Armcx3 endogenous protein has been described as being localized in at least three sub-cellular compartments: mitochondria, nuclei and cytosol. Moreover, the N-terminal region contains a mitochondrial targeting sequence that is necessary and sufficient for the targeting of Armcx3 to mitochondria (López-Doménech et al., 2012). Thus, deletion of the first N-terminal 12 amino acids abolished mitochondrial targeting, leading to nuclear localization (López-Doménech et al., 2012). We wondered whether Armcx3 mitochondrial localization was required to regulate progenitor cell proliferation and differentiation. We thus cloned the Armcx3 deletion mutant [pCIGArmcx3Δ(1-12)] in pCIG vector and electroporated chick embryos as above. First, we analyzed the intracellular localization of both proteins 24hpe with immunohistochemical analysis, using an anti-Armcx3 antibody and the mitochondrial marker anti-COXIV. We confirmed that whereas Armcx3 protein co-localizes with COXIV in mitochondria, the mutant Armcx3Δ(1-12) protein does not exhibit mitochondrial localization, but shows diffuse cytosolic and nuclear staining (Figure 3A).

**Figure 4:**
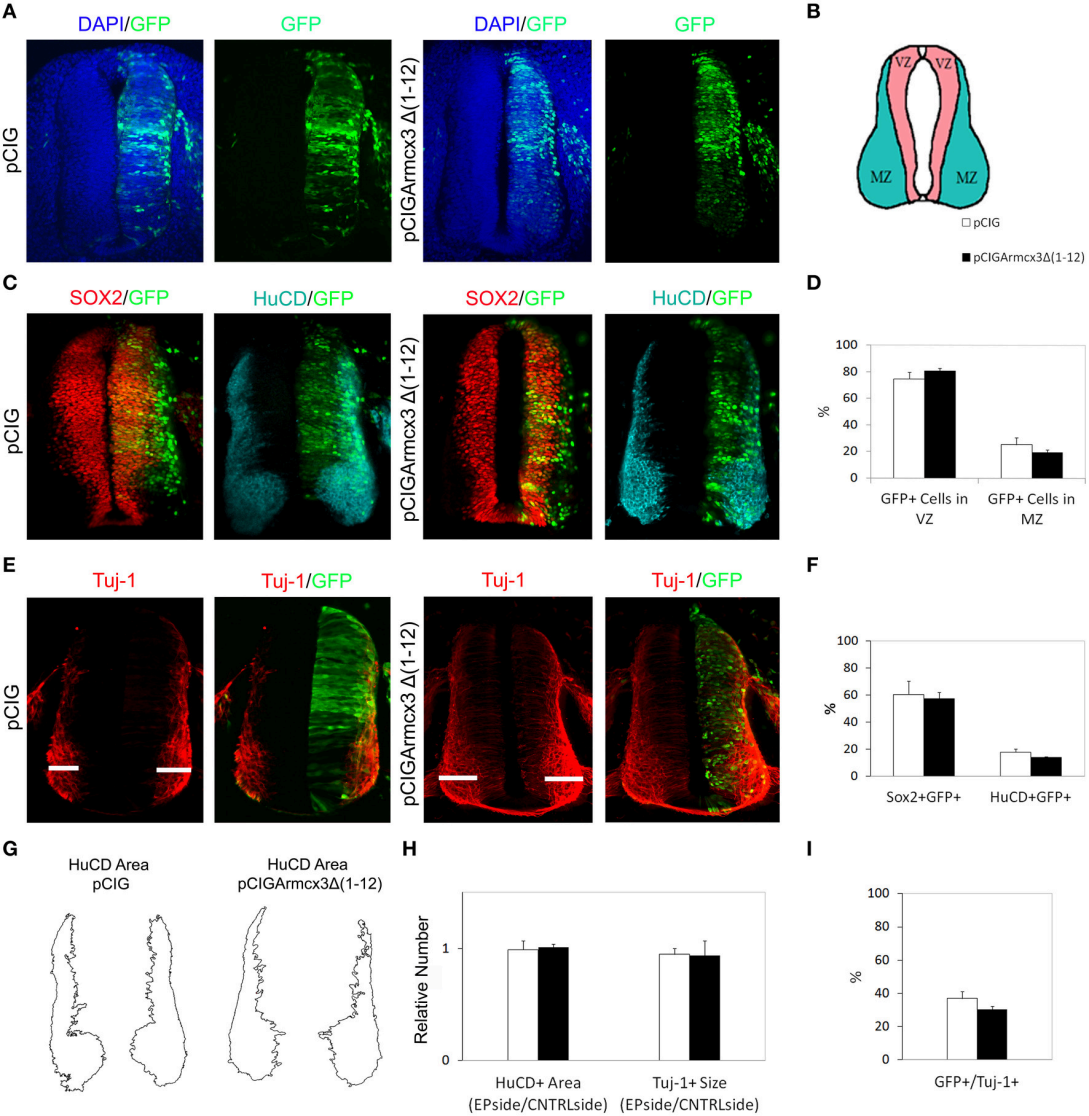
**Armcx3 effects on neural differentiation depend on its mitochondrial localization. (A,C,E)** Representative transverse sections of chick neural tubes from embryos electroporated at HH stage 12 with the indicated plasmids and processed and analyzed 48hpe as described in Figure 2. In contrast to the full length Armcx3 (see Figure 2), the overexpression of a non-mitochondrial truncated version of Arcmx3 (Armcx3Δ1-12) did not induce apparent changes in the lateral distribution of progenitors **(A–D)** or modify the percentage of Sox2/GFP or HuCD/GFP positive cells. **(F)** Similarly, Armcx3Δ1-12 overexpression has no impact on the HuCD + area **(G,H)**, the width of Tuj-1 positive region **(G,H)**, or the percentage of electroporated cells positive for Tuj-1 **(I)**. Data represent the mean ± s.e.m.

We observed that, in contrast to Armcx3-expressing cells, pCIGArmcx3Δ(1-12) electroporated cells showed an even distribution through the MZ and VZ at both 24 and 48hpe, similarly to the control, pCIG-electroporated cells (Figures 3B–E, 4A–D). Similarly, there were no differences between pCIGArmcx3Δ(1-12) and control-electroporated embryos in the percentages of GFP/Sox2^+^cells, GFP/HuC/D^+^ cells (Figures 3E,F, 4C,F), nor in proliferation markers (Figures 3G–I) or neuronal differentiation markers (Tuj-1 and HuC/D, Figures 4E,G–I). Taken together, these data indicate that mitochondrial localization is required for Armcx3 to regulate progenitor proliferation and neuronal differentiation.

### Ectopic Armc10 localizes at mitochondria and its overexpression reduces progenitor proliferation without affecting neuronal maturation

**Figure 5:**
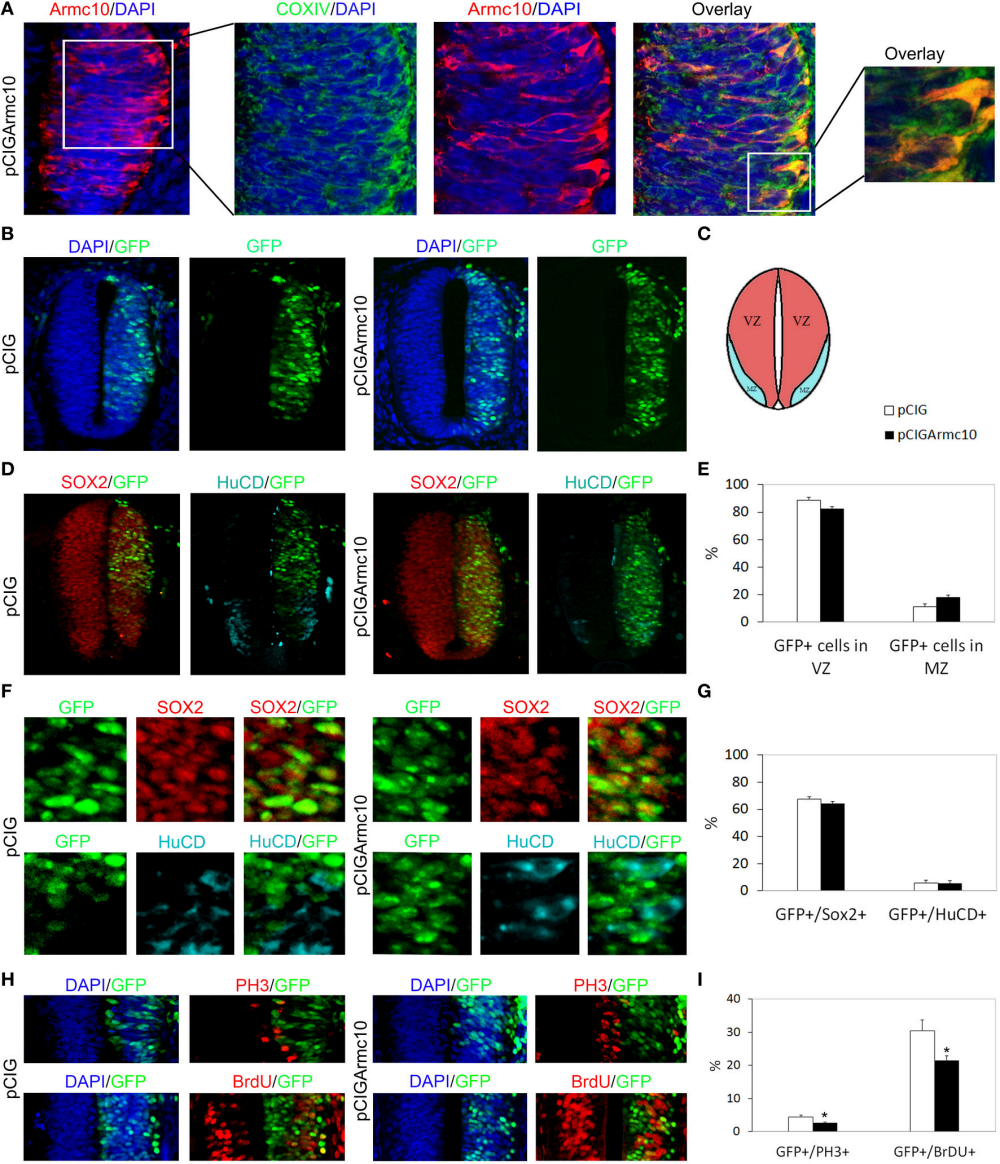
**Armc10 overexpression reduces progenitors proliferation**. Representative transverse sections of chick neural tubes from embryos electroporated at HH stage 12 with the indicated plasmids and processed and analyzed 24hpe as described in Figure 1. As is seen with Arcmx3, Armc10 protein is localized at the mitochondria of neural progenitors as indicated by its colocalization with the mitochondrial marker CoxIV **(A)**. **(C)** Schematic representation illustrating the distributions of the VZ and MZ at HH12+24hpe. Armc10 overexpression did not induce changes in the lateral distribution of electroporated neural progenitors **(B,E)** or in the percentage of GFP/Sox2 or GFP/HuCD positive cells **(D,F,G)**. However, it induced a reduction in the percentage of PH3/GFP and BrdU/GFP positive cells **(H,I)**. Data represent the mean ± s.e.m. (^*^p < 0.05).

Armcx3 belongs to the Eutherian-specific Armcx gene cluster (Armcx1-6, GASP1-3), which arose by retrotransposition from the Armc10 ancestral gene and by subsequent short-range tandem duplications of a rapidly evolving region in the X chromosome (López-Doménech et al., 2012). To investigate possible evolution-related functional differences between Armcx3 and the ancestor gene Armc10, we performed studies similar to those above, in the chicken spinal cord, but overexpressing an Armc10 construct (pCIGArmc10). Similarly to Armcx3, electroporation of pCIGArmc10 in the neural tube yielded a pattern of staining which largely colocalized with the mitochondrial marker COXIV (Figure 5A).

**Figure 6:**
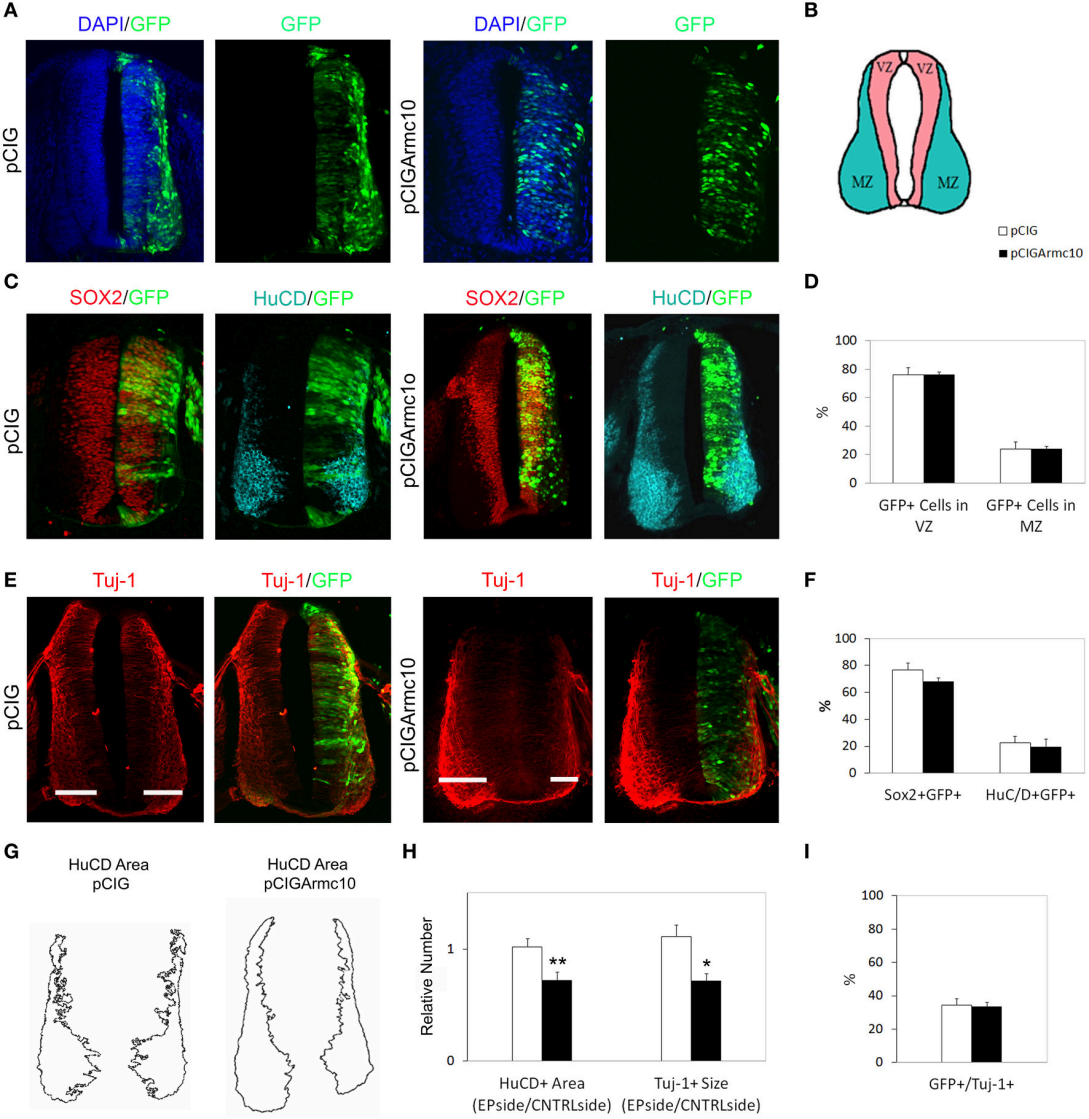
**Armc10 overexpression does not promote neural differentiation**. Representative transverse sections of chick neural tubes from embryos electroporated at HH stage 12 with the indicated plasmids and processed and analyzed 48hpe as described in Figure 2. The overexpression of Armc10 did not induce changes in the lateral distribution of electroporated progenitors **(A)** or modify the percentage of Sox2/GFP or HuCD/GFP positive cells **(C,D)**. By contrast, Armc10 overexpression reduced the HuCD area and the width of Tuj-1 positive region **(G,H)**. The percentage of electroporated cells positive for Tuj-1 was not different in embryos where Armc10 was overexpressed with respect to controls (pCIG) **(I)**. Data represent the mean ± s.e.m. (^*^p < 0.05, ^**^p < 0.01).

We observed that, both at 24 and 48 hpe times, Armc10-expressing cells showed a homogenous distribution throughout the VZ and MZ, in contrast to the distribution found after Armcx3 electroporation (Figures 5B–E, **6A–D**). Moreover, we did not observe changes in the proportion of GFP/Sox2^+^, or GFP/HuC/D^+^cells, compared to controls (Figures 5F,G, 6C,F).

However, we found a significant decrease in proliferation markers in pCIGArmc10-electroporated embryos, 24hpe (Figures 5H,I), indicating that, likeArmcx3, Armc10 is a negative regulator of cell cycle *in vivo*. The reduction in proliferation at early stages of development correlated with a reduction in the width of Tuj-1 and HuC/D-positive regions 48hpe (Figures 6G,H). Nevertheless, we did not find any variation in the proportion of Tuj-1^+^/GFP^+^ cells with respect to the controls, indicating that ectopic Armc10 expression reduces progenitor proliferation without affecting neuronal differentiation (Figures 6E,I).

### Armc10 endogenous gene is expressed at HH12, HH19, and HH24 stages

**Figure 7:**
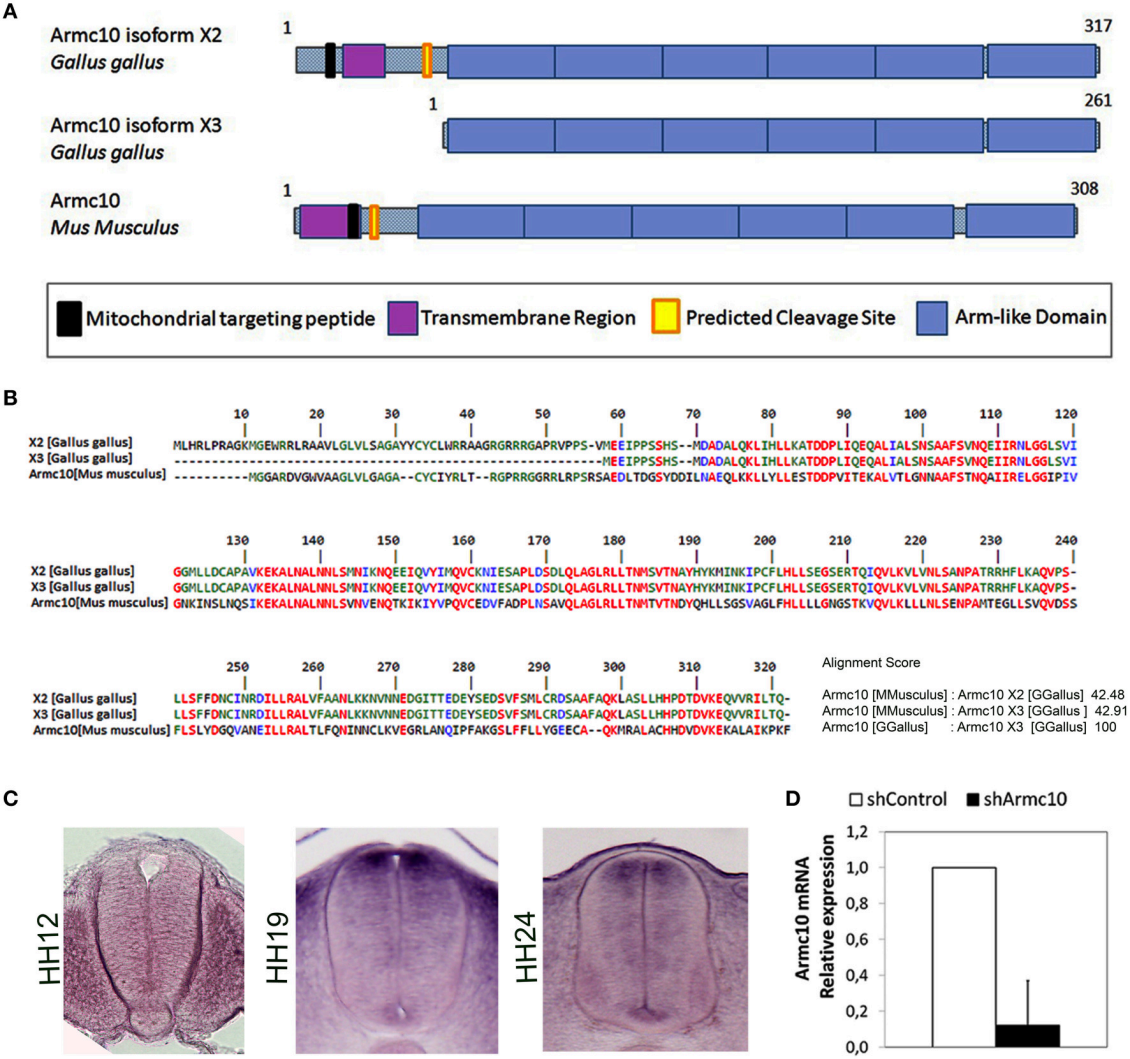
**Endogenous Armc10 is expressed in chicken spinal cord. (A)** Structure and domains of the two different isoforms of chicken Armc10 protein predicted by *in silico* analysis. They differ in the N-terminal region, containing a mitochondrial targeting peptide, an N-terminal transmembrane region, and a predicted cleavage site. The C-terminal domain consists of up to six Arm-like tandem repeats. **(B)** Both Armc10 isoforms share a strong sequence homology with mouse Armc10. **(C)** HH stage 12, 19, and 24 showing expression of chick Armc10. **(D)** Efficiency of Armc10 gene silencing using shRNA in chicken neural tube, as measured with RT-qPCR. Armc10 mRNA expression was calculated relative to that of control shRNA (shControl; error bars indicate the SD).

**Figure 8:**
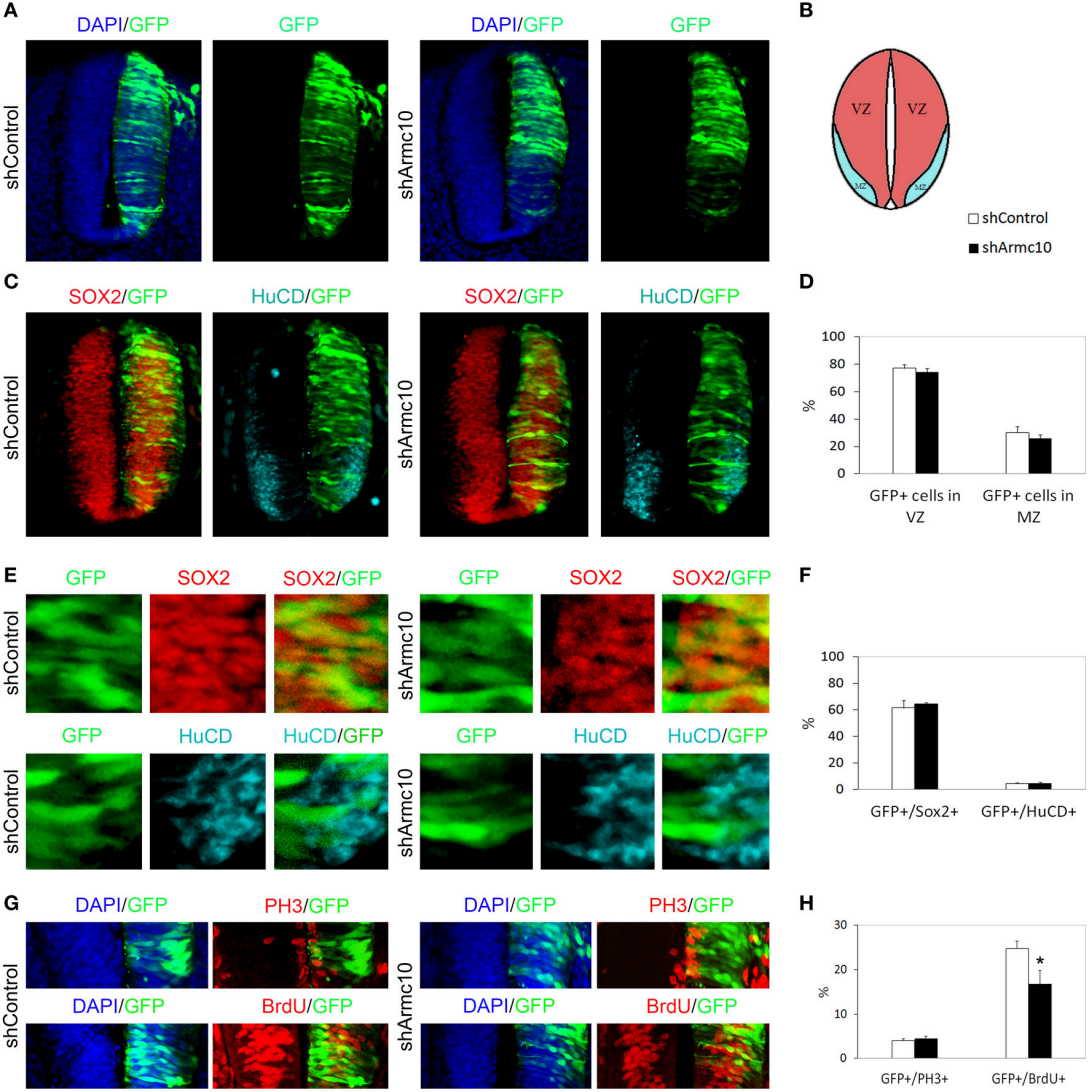
**Endogenous Armc10 silencing inhibits progenitor proliferation. (A–D)** Representative transverse sections of neural tubes from embryos electroporated at HH stage 12 with shControl and shArmc10 vectors and analyzed at 24hpe with the indicated immunostaining. No changes in the distribution of shArmc10 expressing cells are observed. **(E,F)** Armc10 silencing does not affect the percentage of HuC/D/GFP or Sox2/GFP-positive cells with respect to the control. **(G,H)** The percentage of GFP-positive electroporated cells positive for BrDU decreases in shArmc10 electroporated embryos; no changes are observed in the percentage of GFP-positive electroporated cells positive for PH3 with respect to the control. Data represent the mean ± s.e.m. (^*^p < 0.05).

As the Armcx gene cluster is specific to eutherian mammals, we first wanted to characterize the expression of the ancestor gene, Armc10, the only locus present in chick. *In silico* analysis predicted two different isoforms for the Armc10 protein, differing in their N-terminal region which contains a putative transmembrane region (Figure 7A). Both Armc10 chick isoforms share strong sequence homology with mouse Armc10 (Figure 7B). We next characterized the expression of the chick Armc10 using *in situ* hybridization inHH12, HH19 and HH24 embryos. Low expression was found at the HH12 stage. At HH19, Armc10 was expressed preferentially in dorsal regions including the roof plate, and weakly in the floor plate (Figure 8C). By HH24, when neural differentiation was more active, Armc10 was expressed in the VZ, with higher expression at dorsal levels, as well as in the floor plate and in motor neurons (Figure 7C).

In order to test the effect of endogenous Armc10 silencing on neural tube development, we generated two different short RNA hairpin (shRNA)-based expression vectors targeting two different sequences of the Armc10 transcript, also expressing GFP and targeting both Armc10 isoforms. To test silencing efficacy, HH12 embryos were electroporated with Armc10 silencing vectors (used together 1:1, shArmc10) or with a control vector (shControl); GFP positive cells were harvested with FACS (MoFlo flow cytometer) and Armc10 transcripts were measured with RT-PCR, demonstrating a 90% reduction in Armc10-silenced cells (Figure 7D).

### Armc10 silencing inhibits progenitor proliferation without affecting neuronal differentiation

**Figure 9:**
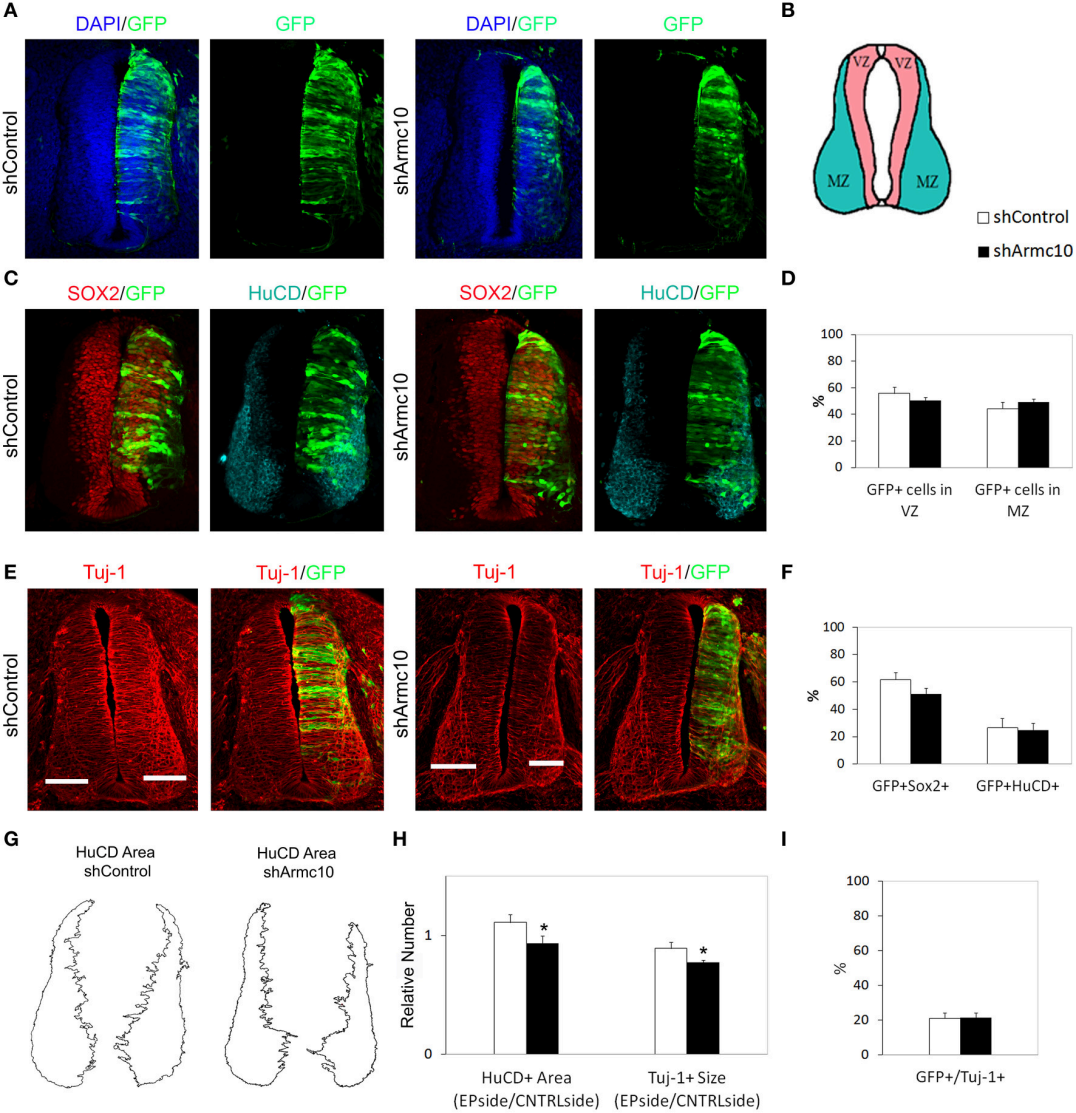
**Endogenous Armc10 silencing inhibits progenitor proliferation but does not affect neural maturation. (A–F)** Representative transverse sections of neural tubes from embryos electroporated at HH stage 12 with shControl and shArmc10 plasmids and analyzed at 48hpe with the indicated immunostaining. No changes in the distribution of shArmc10 expressing cells **(D)** or in the percentage of HuC/D/GFP and Sox2/GFP-positive cells **(F)** are observed (anti-HuC/D, blue; anti-Sox2, red). **(G,H)** The HuC/D+ areas corresponding to the MZ (formed by the differentiating neurons) were defined using ImageJ processing **(G)**. The areas measured for the electroporated side (EP) were standardized to their contralateral controls (CNTRL) and are presented as ratios of the area of MZ (HuC/D^+^Area); the widths of the Tuj-1-marked region for the electroporated side were standardized to their contralateral controls and are presented as ratios of the size of MZ (Tuj^+^ Size) **(H)**. **(I)** Histogram showing the percentage of electroporated cells (GFP^+^) positive for Tuj-1. Data represent the mean ± s.e.m. (^*^p < 0.05).

HH12 embryos were electroporated with shArmc10 or shControl vectors and examined 24 or 48hpe. We did not observe differences in the distribution of GFP^+^ electroporated cells in the mantle zone (Figures 8A–D, 9A–D), or in the proportion of GFP/Sox2, GFP/HuC/D and GFP/Tuj-1 -positive cells (Figures 8C,E,F, 9C,E,F,I). Moreover, we did not find changes in the number of GFP-positive cells expressing PH3, following Armc10 silencing, although we found a 30% decrease in the number of GFP-positive cells incorporating BrdU, suggesting that appropriate levels of Armc10 protein are required to achieve proliferation (Figures 8G,H).

The slight reduction in proliferation at early stages of development also correlated with a reduction in Tuj-1 and HuC/D-positive areas 48hpe (Figures 9E,G,H), suggesting that this change may be a direct consequence of the reduced progenitor proliferation found at 24hpe.

### Armcx3 and Armc10 expression levels may regulate β-catenin-induced Tcf/LEF transcriptional activity without affecting dorsoventral patterning

**Figure 10:**
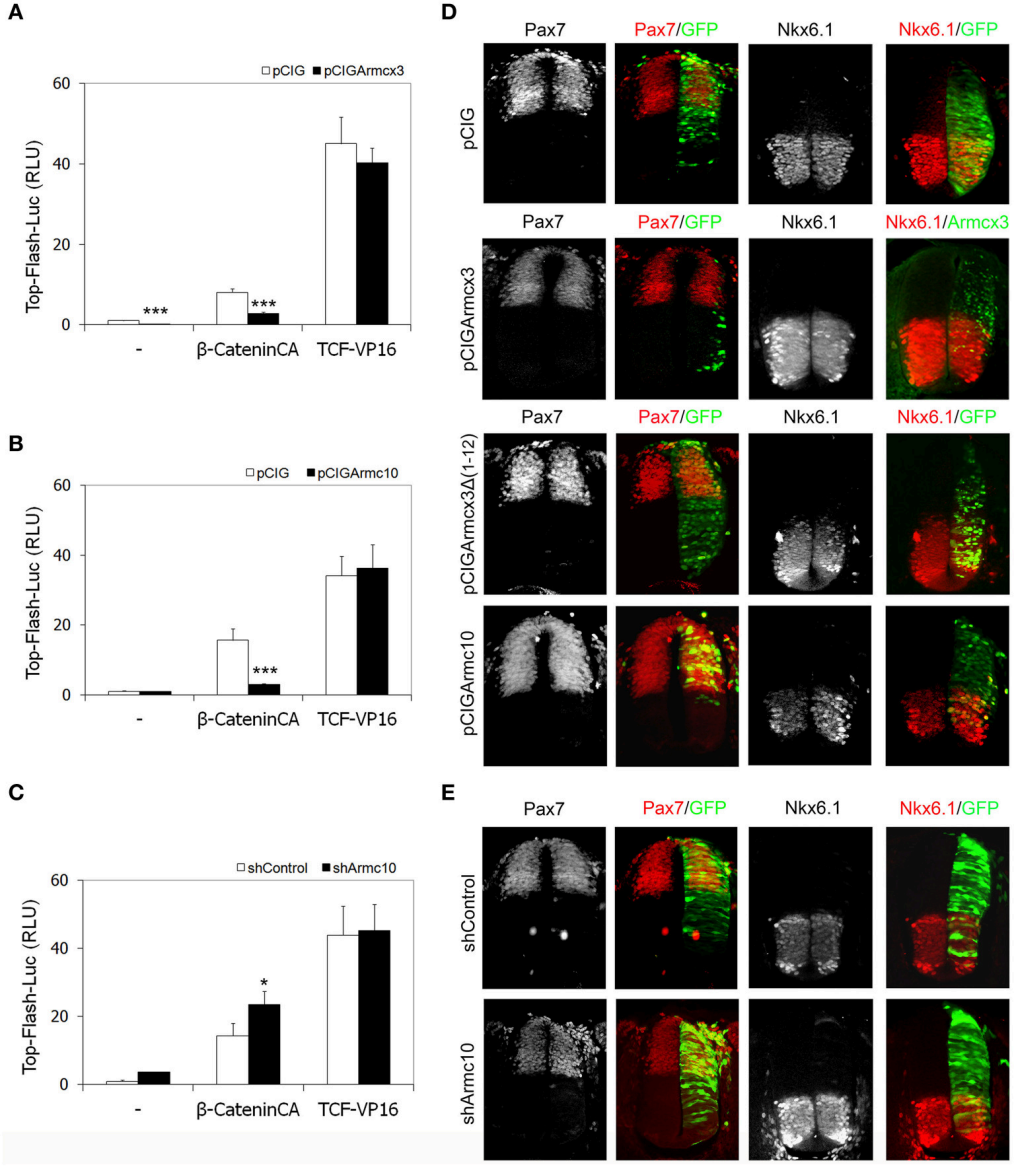
**Armcx3 and Armc10 expression levels regulate β-catenin-induced Tcf/LEF transcriptional activity without affecting dorsoventral patterning. (A,B)** Armcx3 and Armc10 inhibit the Wnt pathway upstream from the TCF activity. HH stage 12 embryos were co-electroporated with the pCIG empty vector, β-catenin^CA^ or TCF-VP16 together with Armcx3 or Armc10. Analysis of the TOP-Flash reporter activity shows that both Armcx3 and Armc10 inhibit β-catenin^CA^-mediated transcriptional activity but not TCF activation. **(C)** Inhibition of endogenous Armc10 activates Wnt pathway. A similar assay electroporating TOP-Flash reporter together with shArmc10 constructs shows an increase in β-catenin^CA^-mediated transcriptional activity. **(D,E)** Alterations in Tcf/LEF transcriptional activity mediated by changes in Armcx3 or Armc10 expression levels do not affect dorsoventral patterning organization. Representative sections of HH12 neural tubes electroporated with the indicated vectors and processed 24hpe for immunostaining analysis against the dorsoventral markers Pax7 or Nk6.1 (red). Anti-GFP or anti-Armcx3 (green) antibodies were used to report the transgene expression. Data represent the mean ± s.e.m. (^*^P < 0.05; ^**^P < 0.01; ^***^P < 0.001).

The Armcx/Armc10 members contain in their sequence several Armadillo-like domains (López-Doménech et al., 2012), and the Wnt-β-catenin pathway favors neural tube progenitor proliferation by directly regulating the transcription of several cell cycle key modulators, such as cyclinD1 and N-myc (Tetsu and McCormick, 1999; Ten Berge et al., 2008). Therefore, to test whether Armcx3 or Armc10 expression levels interfere with the Wnt-β-catenin pathway, we performed *in vivo* luciferase assays measuring Tcf/LEF-transcriptional activity. Chick embryos were electroporated with the TOP-FLASH-Luc reporter together with two different activators of the Tcf/LEF transcriptional activity: a stable form of β-catenin (β-catenin^CA^) and a chimeric transcriptional activator form containing the HMG box DNA-binding domain of Tcf fused to the VP16 transactivator domain (Tcf3-VP16) (Kim et al., 2000). Analysis of luciferase activity 24hpe showed that both Armcx3 and Armc10 expression were able to decrease Tcf/LEF-transcriptional activity at basal conditions as well as following β-cateninCA induction (Figures 10A,B). However, neither Armcx3 nor Armc10 inhibited the Tcf3-VP16- dependent transactivation (Figures 10A,B), suggesting that this regulation takes place upstream from the transcriptional activity.

In contrast, Armc10 silencing was able to activate Tcf/LEF-transcriptional activity at baseline conditions (Figure 10D) as well as following β-catenin^CA^ induction, without inhibiting the Tcf3-VP16- dependent transactivation (Figure 10C).

Interestingly, the regulatory effect on Tcf/LEF activation of Armcx3 and Armc10 overexpression or Armc10 silencing is not associated with changes in dorsoventral patterning determination, assessed by analysis of dorsal marker Pax7, central marker Pax6, and ventral markers Nkx6.1 or Nkx2.2 (Figures 10C,E and data not shown). Further experiments using Cath1/Atoh1, Ngn1, and Cash1/Asch1 (respectively, dP1, dP2, and dP3 markers) did not reveal changes in the electroporated side between control, shArmc10 and Armc10 conditions (Supplementary Figure 2). Moreover, we did not find changes in apoptosis, as assessed by activated caspase 3 expression at 24hpe (Supplementary Figure 3).

## Discussion

In this study we analyzed the function of Armcx/Armc10 proteins during chick neural tube development. Whereas, in mammals several members of the family are described (López-Doménech et al., 2012), the only Armcx/Armc10 member found in chick is the Armc10 gene, producing two isoforms, both sharing a strong protein sequence homology with mouse Armc10. In developing chickens, spinal cord Armc10 transcripts are distributed in a dorsal to ventral gradient (highest dorsally) in mitotically active neural precursors. We found Armc10 transcripts also weakly localized in the floor plate and, at later stages, in motorneurons (Figure 7C). This expression pattern suggests Armc10 participation in the control of NPC proliferation and differentiation programs.

Consistent with this idea, mouse Armc10 overexpression in chicken spinal cord induces a reduction in the proliferation of NPCs (Figures 5D–I). The same effect is observed when murine Armcx3 is overexpressed (Figures 1C–H), suggesting a degree of functional conservation between Armcx3 and its ancestor gene. Compatible with a role of these proteins in regulating cell cycle, several members of Armcx family were initially described as putative tumor-suppressor genes (Kurochkin et al., 2001; Mishra et al., 2011; Tuszynski et al., 2011; Iseki H et al., 2012). Finally, counts of BrdU-positive cells among non-electroporated (GFP-) cells surrounding GFP-positive cells did not reveal differences among distinct experimental groups (Supplementary Figure 1), suggesting that the effects of Armcx3/Armc10 dysregulation are cell autonomous. Further studies are required to establish whether the negative action of Armc10 and Armcx3 on NPC cell cycle is achieved by affecting cell cycle length, inducing an early cell cycle exit, or by altering the balance between the three modes of division in neural precursors (PP, PN, NN), (Lui et al., 2011; Franco and Muller, 2013).

By contrast with Armc10, Armcx3 overexpression promotes neural differentiation, a conclusion supported by the fact thatArmcx3 (GFP^+^) electroporated cells are mainly located at the MZ of the neural tube. Moreover, most Armcx3 electroporated cells are Tuj-1 or HuC/D positive, producing a significant increase in the percentage of Tuj-1^+^/GFP^+^ or HuC/D/GFP^+^ cells with respect to the control embryos (Figures 2C–F,H). These differences between the effects due to Armcx3 or Armc10 overexpression during spinal cord development highlight functional divergence between the two proteins, and suggest that eutherian Armcx proteins may have acquired additional functions with respect to the ancestor Armc10 gene. The extent of the functional divergences between Armcx/Armc10 family members through evolution is an interesting issue to be clarified in the future.

The mechanisms by which Armcx proteins participate in the regulation of NPC cell proliferation/differentiation remain to be discovered. It is possible that the Armcx3 or Armc10 regulation of proliferation/differentiation balance during spinal cord development originates in their role in regulating mitochondrial function, morphology and localization (López-Doménech et al., 2012; Serrat et al., 2014). This notion is consistent with our data showing that the overexpression of an Armcx3 truncated form, which lacks its mitochondrial localization signal, does not produce any alterations in the NPC proliferation or differentiation. However, it remains to be investigated whether Armc10 mitochondrial targeting is also required for Armc10 effects in the spinal cord. It is well known that mitochondria must undergo morphological changes and be distributed properly in cells that are dividing (Lee et al., 2007; Mitra et al., 2009). The morphology of mitochondria is primarily controlled by fusion and fission events and it has been shown that several proteins regulating these processes, such as Mitofusin2, Drp1, and Miro1, also play a regulatory role in the cell cycle (Mitra et al., 2009, 2012; Yamaoka et al., 2011; Chen et al., 2014; Martorell-Riera et al., 2015). Moreover, the reorganization of mitochondria during mitosis is accompanied with detachment of mitochondria from microtubules and dynein motor complex (Lee et al., 2005). It has been demonstrated that both Armcx3 and Armc10 cause mitochondrial aggregation and/or tethering in HEK293 cells and hippocampal neurons, where these processes are believed to serve to capture mitochondria at specific locations (Chang and Reynolds, 2006; MacAskill and Kittler, 2010). Moreover, Armcx3 and Armc10 interact with the Kinesin5/Miro/Trak2 complex, controlling mitochondrial trafficking along microtubules in neurons, through a direct interaction with Miro and Trak2 proteins (López-Doménech et al., 2012; Serrat et al., 2014).

Armcx/Armc10 mitochondrial related regulation of NPC proliferation may explain our findings where the endogenous Armc10 downregulation, performed by silencing both Armc10 isoforms, also causes a reduction in the proliferation of NPCs, without affecting the neural differentiation process (Figures 8, 9). Thus, it has been described how both Armcx3 and Armc10 gain and loss-of function induce similar phenotypes related with mitochondrial trafficking regulation (López-Doménech et al., 2012; Serrat et al., 2014). This effect has also been described for TDP43 and other mitochondria-targeted proteins (Wang et al., 2013; Magrané et al., 2014). Altogether, our results suggest a model in which appropriate levels of Armc10 protein are essential to guarantee normal progression through the cell cycle.

Armcx3 has been shown to interact with transcription factor Sox10 and increase its outer membrane of mitochondrial localization (Mou et al., 2009). Sox10 is involved in several processes during CNS development, including differentiation (Herbarth et al., 1998) and specification of neural crest-derived sensory neurons (Elworthy et al., 2003, 2005; Carney et al., 2006). It has been proposed that the interaction between Armcx3 and Sox10 within the cytoplasm could lead to post-translational modifications of Sox10 resulting in increased transcriptional activity once it is transported into the nucleus (Mou et al., 2009). Further study of the participation of Armcx3/Armc10 in regulating the activity of Sox family transcription factors could open new perspectives in understanding the molecular mechanisms by which these proteins regulate developmental processes.

In order to find a molecular pathway via which Armcx3 and Armc10 may carry out their function during development, we considered their protein structure: all members of the Armcx/Armc10 cluster code for proteins containing six armadillo repeats, found in a wide range of proteins related to Wnt/β-catenin signaling, such as β-catenin or Adenomatous polyposis coli (APC). In addition, Armc10 expression pattern in chicken spinal cord resembles the expression of the mitogenic Wnt proteins (composed principally of Wnt1 and Wnt3a) across the VZ of the neural tube (Megason and McMahon, 2002). The Wnt/β-catenin signaling pathway plays a pivotal role in spinal cord development, for instance by regulating patterning of the neural tube, promoting the proliferation of dorsal spinal progenitor cells, and inducing the differentiation of dorsal spinal neurons. Thus, although further experiments are required, our data suggest that both Armc10 and Armcx3 may inhibit the canonical Wnt/β-catenin pathway and that their inhibition takes place upstream from the transcriptional activity (Figures 10A,B). Moreover, Armc10 silencing is able to activate Tcf/LEF-transcriptional activity (Figure 10C). These data suggest that the effects of altered Armc10 expression levels on NPC proliferation cannot be exclusively ascribed to Armc10's inhibitory function in the Wnt/β-catenin pathway; indeed, both Armc10 overexpression and Armc10 silencing induce the same effect on NPC proliferation, indicating that other mechanisms may be involved in combination with Wnt/β-catenin pathway modulation. Supporting this notion, we have not observed changes in spinal cord patterning upon Armcx3 and Armc10 gain or loss of function manipulations (Figure 10D).

Taken together, our data show that Armcx3 and Armc10 proteins can regulate key processes of spinal cord development. We postulate that Armcx3 and Armc10 represent crucial elements coordinating mitochondrial distribution and partitions during NPC cell cycle progression. Consequentially, altered expression levels of these proteins affect the NPC proliferation process and in turn neural maturation. Nevertheless, a possible mechanism by which Armcx3 and Armc10 could control proliferation process through mitochondrial dynamics regulation remains to be further clarified. The identification of the molecular mechanism involved in Armcx protein action is one of the most stimulating challenges of the near future.

## Author contributions

Conceived and designed the experiments: ES, SM, FU, EM. Performed the experiments: SM, FU, IG. Analyzed the data: ES, SM, FU, EM. Wrote the paper: SM, ES, FU.

## Funding

This work was supported by grants from Spanish MINECO (SAF2013-42445R), CIBERNED (ISCIII) and La Marató de TV3 Foundation to ES; Spanish MINECO (BFU2010-21507) to FU; BFU2013-46477-P to EM.

### Conflict of interest statement

The authors declare that the research was conducted in the absence of any commercial or financial relationships that could be construed as a potential conflict of interest.

## Supplementary material

Supplementary Figure 1 **Armcx3 and Armc10 effects on NPC proliferation are cell autonomous**. Quantification of the percentage of BrdU+ cells among non-electroporated (GFP-) cells surrounding GFP + transfected cells in HH12 chick embryos electroporated with the indicated constructs and analyzed at 24 h PE. No differences between the different experimental conditions can be appreciated suggesting that the effects of Armcx3/Armc10 dysregulation on NPC proliferation are cell autonomous.

Supplementary Figure 2 **Armc10 expression levels do not regulate the expression of proneural dorsal genes**. Loss- and gain-of-function experiments in chick embryos reveal that the expression of bHLH proneural genes is not altered by Armc10 expression levels. The electroporated side is to the right (indicated as+). The expression of Cath1/Atoh1 **(A–C)**, Ngn1 **(D–F)**, and Cash1/Asch1 **(G–I)**, were analyzed in HH12 embryos electroporated with an empty control vector, and with sh-Armc10 and pCIGArmc10 vectors, at 36 h PE.

Supplementary Figure 3 **Alteration in Armcx3 and Armc10 expression levels does not induce apoptosis**. Transverse sections of neural tubes from chick embryos electroporated at HH stage 12 with the indicated vectors. Anti-GFP or anti-Armcx3 (green) antibodies were used to report the transgene expression; anti-activated caspase3 antibody was used to detect apoptotic cells (red). Gli3R was used as positive control of apoptosis.

## References

[B1] Alvarez-Medina R. Le Dreau G. Ros M. Marti E. (2009). Hedgehog activation is required upstream of Wnt signalling to control neural progenitor proliferation. Development 136, 3301–3309. 10.1242/dev.041772 19736325

[B2] Behrens J. Von Kries J. P. Kühl M. Bruhn L. Wedlich D. Grosschedl R.. (1996). Functional interaction of beta-catenin with the transcription factor LEF-1. Nature 382, 638–642. 10.1038/382638a0 8757136

[B3] Bluske K. K. Vue T. Y. Kawakami Y. Taketo M. M. Yoshikawa K. Johnson J. E.. (2012). beta-Catenin signaling specifies progenitor cell identity in parallel with Shh signaling in the developing mammalian thalamus. Development 139, 2692–2702. 10.1242/dev.072314 22745311 PMC3392701

[B4] Bowman A. N. van Amerongen R. Palmer T. D. Nusse R. (2013). Lineage tracing with Axin2 reveals distinct developmental and adult populations of Wnt/beta-catenin-responsive neural stem cells. Proc. Natl. Acad. Sci. U.S.A. 110, 7324–7329. 10.1073/pnas.1305411110 23589866 PMC3645553

[B5] Carney T. J. Dutton K. A. Greenhill E. Delfino-Machin M. Dufourcq P. Blader P.. (2006). A direct role for Sox10 in specification of neural crest-derived sensory neurons. Development 133, 4619–4630. 10.1242/dev.02668 17065232

[B6] Cayuso J. Ulloa F. Cox B. Briscoe J. Martí E. (2006). The Sonic hedgehog pathway independently controls the patterning, proliferation and survival of neuroepithelial cells by regulating Gli activity. Development 133, 517–528. 10.1242/dev.02228 16410413

[B7] Chang D. T. Reynolds I. J. (2006). Differences in mitochondrial movement and morphology in young and mature primary cortical neurons in culture. Neuroscience 141, 727–736. 10.1016/j.neuroscience.2006.01.034 16797853

[B8] Chen K. H. Dasgupta A. Ding J. Indig F. E. Ghosh P. Longo D. L. (2014). Role of mitofusin 2 (Mfn2) in controlling cellular proliferation. FASEB J. 28, 382–394. 10.1096/fj.13-230037 24081906 PMC3868832

[B9] Chenn A. Walsh C. A. (2002). Regulation of cerebral cortical size by control of cell cycle exit in neural precursors. Science 297, 365–369. 10.1126/science.1074192 12130776

[B10] Dall'Era M. A. Oudes A. Martin D. B. Liu A. Y. (2007). HSP27 and HSP70 interact with CD10 in C4-2 prostate cancer cells. Prostate 67, 714–721. 10.1002/pros.20558 17342744

[B11] Elworthy S. Lister J. A. Carney T. J. Raible D. W. Kelsh R. N. (2003). Transcriptional regulation of mitfa accounts for the sox10 requirement in zebrafish melanophore development. Development 130, 2809–2818. 10.1242/dev.00461 12736222

[B12] Elworthy S. Pinto J. P. Pettifer A. Cancela M. L. Kelsh R. N. (2005). Phox2b function in the enteric nervous system is conserved in zebrafish and is sox10-dependent. Mech. Dev. 122, 659–669. 10.1016/j.mod.2004.12.008 15817223

[B13] Franco S. J. Müller U. (2013). Shaping our minds: stem and progenitor cell diversity in the mammalian neocortex. Neuron 77, 19–34. 10.1016/j.neuron.2012.12.022 23312513 PMC3557841

[B14] Hamburger V. Hamilton H. L. (1951). A series of normal stages in the development of the chick embryo. J. Morphol. 88, 49–92. 10.1002/jmor.1050880104 24539719

[B15] Han Y. G. Spassky N. Romaguera-Ros M. Garcia-Verdugo J. M. Aguilar A. Schneider-Maunoury S.. (2008). Hedgehog signaling and primary cilia are required for the formation of adult neural stem cells. Nat. Neurosci. 11, 277–284. 10.1038/nn2059 18297065

[B16] Hart M. Concordet J. P. Lassot I. Albert I. del Los Santos R. Durand H.. (1999). The F-box protein beta-TrCP associates with phosphorylated beta-catenin and regulates its activity in the cell. Curr. Biol. 9, 207–210. 10.1016/S0960-9822(99)80091-8 10074433

[B17] Herbarth B. Pingault V. Bondurand N. Kuhlbrodt K. Hermans-Borgmeyer I. Puliti A.. (1998). Mutation of the Sry-related Sox10 gene in Dominant megacolon, a mouse model for human Hirschsprung disease. Proc. Natl. Acad. Sci. U.S.A. 95, 5161–5165. 10.1073/pnas.95.9.5161 9560246 PMC20231

[B18] Hirabayashi Y. Itoh Y. Tabata H. Nakajima K. Akiyama T. Masuyama N.. (2004). The Wnt/beta-catenin pathway directs neuronal differentiation of cortical neural precursor cells. Development 131, 2791–2801. 10.1242/dev.01165 15142975

[B19] Iseki H. Takeda A. Andoh T. Kuwabara K. Takahashi N. Kurochkin I. V.. (2012). ALEX1 suppresses colony formation ability of human colorectal carcinoma cell lines. Cancer Sci. 103, 1267–1271. 10.1111/j.1349-7006.2012.02300.x 22494058 PMC7659355

[B20] Iseki H. Takeda A. Andoh T. Takahashi N. Kurochkin I. V. Yarmishyn A.. (2010). Human Arm protein lost in epithelial cancers, on chromosome X 1 (ALEX1) gene is transcriptionally regulated by CREB and Wnt/beta-catenin signaling. Cancer Sci. 101, 1361–1366. 10.1111/j.1349-7006.2010.01541.x 20398052 PMC11159271

[B21] Jacinto F. V. Ballestar E. Ropero S. Esteller M. (2007). Discovery of epigenetically silenced genes by methylated DNA immunoprecipitation in colon cancer cells. Cancer Res. 67, 11481–11486. 10.1158/0008-5472.CAN-07-2687 18089774

[B22] Jessen J. R. (2009). Noncanonical Wnt signaling in tumor progression and metastasis. Zebrafish 6, 21–28. 10.1089/zeb.2008.0571 19292672

[B23] Kalani M. Y. Cheshier S. H. Cord B. J. Bababeygy S. R. Vogel H. Weissman I. L.. (2008). Wnt-mediated self-renewal of neural stem/progenitor cells. Proc. Natl. Acad. Sci. U.S.A. 105, 16970–16975. 10.1073/pnas.0808616105 18957545 PMC2575225

[B24] Kim K. Pang K. M. Evans M. Hay E. D. (2000). Overexpression of beta-catenin induces apoptosis independent of its transactivation function with LEF-1 or the involvement of major G1 cell cycle regulators. Mol. Biol. Cell 11, 3509–3523. 10.1091/mbc.11.10.3509 11029052 PMC15010

[B25] Korinek V. Barker N. Willert K. Molenaar M. Roose J. Wagenaar G.. (1998). Two members of the Tcf family implicated in Wnt/beta-catenin signaling during embryogenesis in the mouse. Mol. Cell. Biol. 18, 1248–1256. 10.1128/MCB.18.3.1248 9488439 PMC108837

[B26] Kurochkin I. V. Yonemitsu N. Funahashi S. I. Nomura H. (2001). ALEX1, a novel human armadillo repeat protein that is expressed differentially in normal tissues and carcinomas. Biochem. Biophys. Res. Commun. 280, 340–347. 10.1006/bbrc.2000.4125 11162520

[B27] Le Dréau G. Saade M. Gutiérrez-Vallejo I. Martí E. (2014). The strength of SMAD1/5 activity determines the mode of stem cell division in the developing spinal cord. J. Cell Biol. 204, 591–605. 10.1083/jcb.201307031 24515346 PMC3926951

[B28] Lee S. Kim S. Sun X. Lee J. H. Cho H. (2007). Cell cycle-dependent mitochondrial biogenesis and dynamics in mammalian cells. Biochem. Biophys. Res. Commun. 357, 111–117. 10.1016/j.bbrc.2007.03.091 17400185

[B29] Lee W. L. Kaiser M. A. Cooper J. A. (2005). The offloading model for dynein function: differential function of motor subunits. J. Cell Biol. 168, 201–207. 10.1083/jcb.200407036 15642746 PMC2171595

[B30] Lie D. C. Colamarino S. A. Song H. J. Désiré L. Mira H. Consiglio A.. (2005). Wnt signalling regulates adult hippocampal neurogenesis. Nature 437, 1370–1375. 10.1038/nature04108 16251967

[B31] Lobjois V. Bel-Vialar S. Trousse F. Pituello F. (2008). Forcing neural progenitor cells to cycle is insufficient to alter cell-fate decision and timing of neuronal differentiation in the spinal cord. Neural Dev. 3, 4. 10.1186/1749-8104-3-4 18271960 PMC2265710

[B32] López-Doménech G. Serrat R. Mirra S. D'Aniello S. Somorjai I. Abad A.. (2012). The Eutherian Armcx genes regulate mitochondrial trafficking in neurons and interact with Miro and Trak2. Nat. Commun. 3, 814. 10.1038/ncomms1829 22569362

[B33] Lui J. H. Hansen D. V. Kriegstein A. R. (2011). Development and evolution of the human neocortex. Cell 146, 18–36. 10.1016/j.cell.2011.06.030 21729779 PMC3610574

[B34] Lyashenko N. Winter M. Migliorini D. Biechele T. Moon R. T. Hartmann C. (2011). Differential requirement for the dual functions of beta-catenin in embryonic stem cell self-renewal and germ layer formation. Nat. Cell Biol. 13, 753–761. 10.1038/ncb2260 21685890 PMC3130149

[B35] MacAskill A. F. Kittler J. T. (2010). Control of mitochondrial transport and localization in neurons. Trends Cell Biol. 20, 102–112. 10.1016/j.tcb.2009.11.002 20006503

[B36] MacDonald B. T. Tamai K. He X. (2009). Wnt/beta-catenin signaling: components, mechanisms, and diseases. Dev. Cell 17, 9–26. 10.1016/j.devcel.2009.06.016 19619488 PMC2861485

[B37] Magrané J. Cortez C. Gan W. B. Manfredi G. (2014). Abnormal mitochondrial transport and morphology are common pathological denominators in SOD1 and TDP43 ALS mouse models. Hum. Mol. Genet. 23, 1413–1424. 10.1093/hmg/ddt528 24154542 PMC3929084

[B38] Martorell-Riera A. Segarra-Mondejar M. Reina M. Martínez-Estrada O. M. Soriano F. X. (2015). Mitochondrial fragmentation in excitotoxicity requires ROCK activation. Cell Cycle 14, 1365–1369. 10.1080/15384101.2015.1022698 25789413 PMC4612563

[B39] McDonald S. L. Silver A. (2009). The opposing roles of Wnt-5a in cancer. Br. J. Cancer 101, 209–214. 10.1038/sj.bjc.6605174 19603030 PMC2720208

[B40] Megason S. G. McMahon A. P. (2002). A mitogen gradient of dorsal midline Wnts organizes growth in the CNS. Development 129, 2087–2098. 11959819 10.1242/dev.129.9.2087

[B41] Mishra P. J. Ha L. Rieker J. Sviderskaya E. V. Bennett D. C. Oberst M. D.. (2011). Dissection of RAS downstream pathways in melanomagenesis: a role for Ral in transformation. Oncogene 29, 2449–2456. 10.1038/onc.2009.521 20118982 PMC3287039

[B42] Mitra K. Rikhy R. Lilly M. Lippincott-Schwartz J. (2012). DRP1-dependent mitochondrial fission initiates follicle cell differentiation during *Drosophila oogenesis*. J. Cell Biol. 197, 487–497. 10.1083/jcb.201110058 22584906 PMC3352947

[B43] Mitra K. Wunder C. Roysam B. Lin G. Lippincott-Schwartz J. (2009). A hyperfused mitochondrial state achieved at G1-S regulates cyclin E buildup and entry into S phase. Proc. Natl. Acad. Sci. U.S.A. 106, 11960–11965. 10.1073/pnas.0904875106 19617534 PMC2710990

[B44] Mizutani K. Saito T. (2005). Progenitors resume generating neurons after temporary inhibition of neurogenesis by Notch activation in the mammalian cerebral cortex. Development 132, 1295–1304. 10.1242/dev.01693 15750183

[B45] Montavon C. Gloss B. S. Warton K. Barton C. A. Statham A. L. Scurry J. P.. (2012). Prognostic and diagnostic significance of DNA methylation patterns in high grade serous ovarian cancer. Gynecol. Oncol. 124, 582–588. 10.1016/j.ygyno.2011.11.026 22115852

[B46] Mou Z. Tapper A. R. Gardner P. D. (2009). The armadillo repeat-containing protein, ARMCX3, physically and functionally interacts with the developmental regulatory factor Sox10. J. Biol. Chem. 284, 13629–13640. 10.1074/jbc.M901177200 19304657 PMC2679464

[B47] Nusse R. Fuerer C. Ching W. Harnish K. Logan C. Zeng A.. (2008). Wnt signaling and stem cell control. Cold Spring Harb. Symp. Quant. Biol. 73, 59–66. 10.1101/sqb.2008.73.035 19028988

[B48] Otero J. J. Fu W. Kan L. Cuadra A. E. Kessler J. A. (2004). Beta-catenin signaling is required for neural differentiation of embryonic stem cells. Development 131, 3545–3557. 10.1242/dev.01218 15262888

[B49] Rohrbeck A. Borlak J. (2009). Cancer genomics identifies regulatory gene networks associated with the transition from dysplasia to advanced lung adenocarcinomas induced by c-Raf-1. PLoS ONE 4:e7315. 10.1371/journal.pone.0007315 19812696 PMC2754338

[B50] Rosales-Reynoso M. A. Ochoa-Hernández A. B. Aguilar-Lemarroy A. Jave-Suárez L. F. Troyo-Sanromán R. Barros-Núñez P. (2010). Gene expression profiling identifies WNT7A as a possible candidate gene for decreased cancer risk in fragile X syndrome patients. Arch. Med. Res. 41, 110–118.e112. 10.1016/j.arcmed.2010.03.001 20470940

[B51] Saade M. Gutiérrez-Vallejo I. Le Dréau G. Rabadán M. A. Miguez D. G. Buceta J.. (2013). Sonic hedgehog signaling switches the mode of division in the developing nervous system. Cell Rep. 4, 492–503. 10.1016/j.celrep.2013.06.038 23891002

[B52] Serrat R. Mirra S. Figueiro-Silva J. Navas-Pérez E. Quevedo M. López-Doménech G.. (2014). The Armc10/SVH gene: genome context, regulation of mitochondrial dynamics and protection against Abeta-induced mitochondrial fragmentation. Cell Death Dis. 5, e1163. 10.1038/cddis.2014.121 24722288 PMC5424104

[B53] Ten Berge D. Brugmann S. A. Helms J. A. Nusse R. (2008). Wnt and FGF signals interact to coordinate growth with cell fate specification during limb development. Development 135, 3247–3257. 10.1242/dev.023176 18776145 PMC2756806

[B54] Tetsu O. McCormick F. (1999). Beta-catenin regulates expression of cyclin D1 in colon carcinoma cells. Nature 398, 422–426. 10.1038/18884 10201372

[B55] Tuszynski G. P. Rothman V. L. Zheng X. Gutu M. Zhang X. Chang F. (2011). G-protein coupled receptor-associated sorting protein 1 (GASP-1), a potential biomarker in breast cancer. Exp. Mol. Pathol. 91, 608–613. 10.1016/j.yexmp.2011.06.015 21791203

[B56] Wang I. F. Tsai K. J. Shen C. K. (2013). Autophagy activation ameliorates neuronal pathogenesis of FTLD-U mice: a new light for treatment of TARDBP/TDP-43 proteinopathies. Autophagy 9, 239–240. 10.4161/auto.22526 23108236 PMC3552888

[B57] Xie Z. Chen Y. Li Z. Bai G. Zhu Y. Yan R.. (2011). Smad6 promotes neuronal differentiation in the intermediate zone of the dorsal neural tube by inhibition of the Wnt/beta-catenin pathway. Proc. Natl. Acad. Sci. U.S.A. 108, 12119–12124. 10.1073/pnas.1100160108 21730158 PMC3141926

[B58] Yamaoka S. Nakajima M. Fujimoto M. Tsutsumi N. (2011). MIRO1 influences the morphology and intracellular distribution of mitochondria during embryonic cell division in Arabidopsis. Plant Cell Rep. 30, 239–244. 10.1007/s00299-010-0926-5 20931334

[B59] Yao H. Ashihara E. Maekawa T. (2011). Targeting the Wnt/beta-catenin signaling pathway in human cancers. Expert Opin. Ther. Targets 15, 873–887. 10.1517/14728222.2011.577418 21486121

[B60] Zechner D. Fujita Y. Hulsken J. Müller T. Walther I. Taketo M. M.. (2003). beta-Catenin signals regulate cell growth and the balance between progenitor cell expansion and differentiation in the nervous system. Dev. Biol. 258, 406–418. 10.1016/S0012-1606(03)00123-4 12798297

[B61] Zechner E. L. Bailey M. J. (2004). The Horizontal Gene Pool: an ESF workshop summary. Plasmid 51, 67–74. 10.1016/j.plasmid.2004.01.002 15068024

[B62] Zeller E. Mock K. Horn M. Colnot S. Schwarz M. Braeuning A. (2012). Dual-specificity phosphatases are targets of the Wnt/beta-catenin pathway and candidate mediators of beta-catenin/Ras signaling interactions. Biol. Chem. 393, 1183–1191. 10.1515/hsz-2012-0130 23089536

[B63] Zhou X. Yang G. Huang R. Chen X. Hu G. (2007). SVH-B interacts directly with p53 and suppresses the transcriptional activity of p53. FEBS Lett. 581, 4943–4948. 10.1016/j.febslet.2007.09.025 17904127

