# Supplementary figures and images for: Function of Armcx3 and Armc10/SVH Genes in the Regulation of Progenitor Proliferation and Neural Differentiation in the Chicken Spinal Cord
Source: Front Cell Neurosci. 2016 Mar 3;10:47. doi: 10.3389/fncel.2016.00047 (PMC4776218; doi:10.3389/fncel.2016.00047)

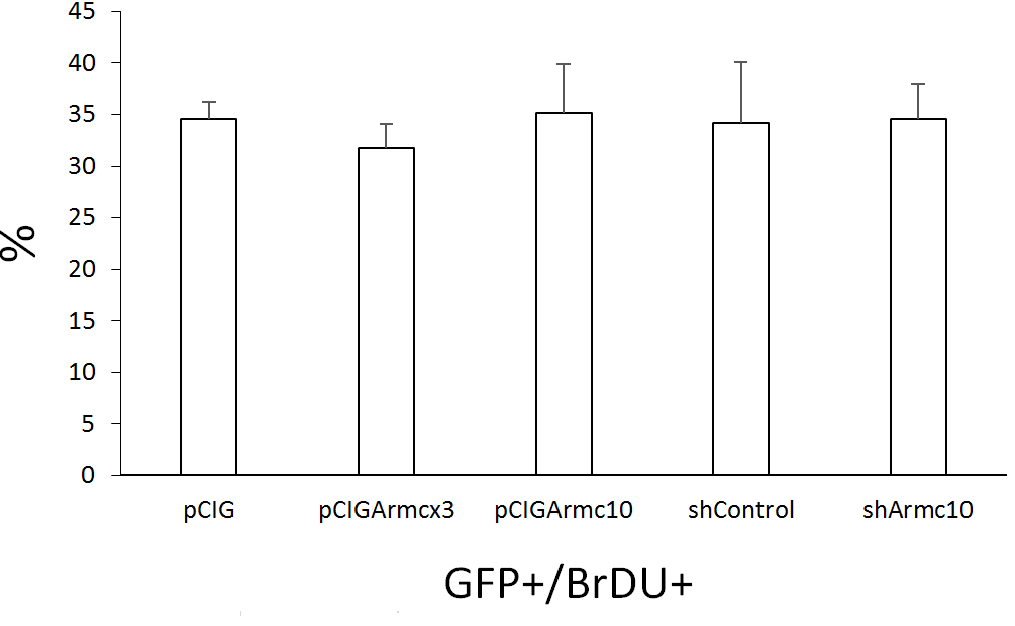

Supplement: Supplementary Figure 1 — Armcx3 and Armc10 effects on NPC proliferation are cell autonomous. Quantification of the percentage of BrdU+ cells among non-electroporated (GFP-) cells surrounding GFP + transfected cells in HH12 chick embryos electroporated with the indicated constructs and analyzed at 24 h PE. No differences between the different experimental conditions can be appreciated suggesting that the effects of Armcx3/Armc10 dysregulation on NPC proliferation are cell autonomous. [file Image1.JPEG]

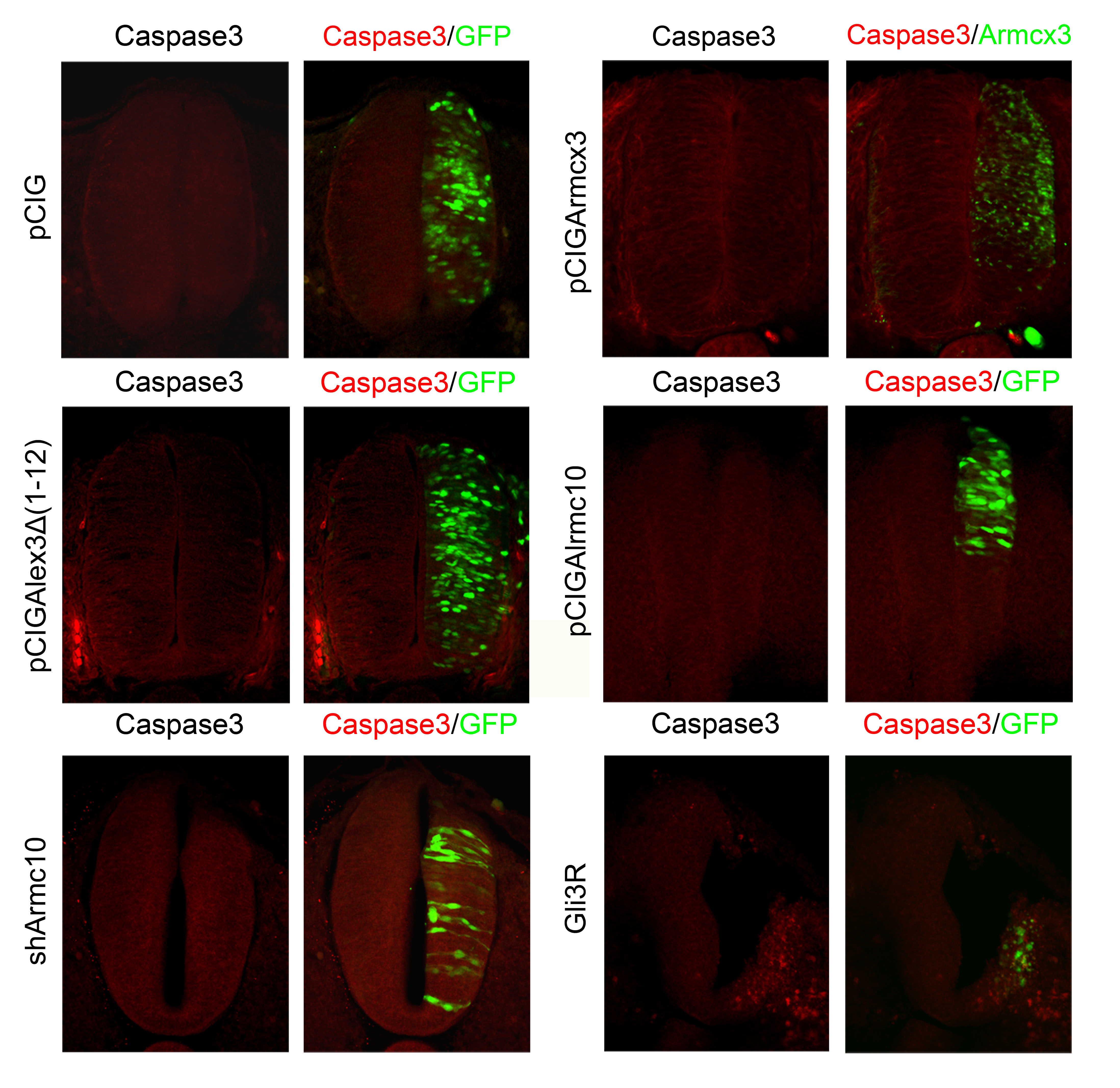

Supplement: Supplementary Figure 3 — Alteration in Armcx3 and Armc10 expression levels does not induce apoptosis. Transverse sections of neural tubes from chick embryos electroporated at HH stage 12 with the indicated vectors. Anti-GFP or anti-Armcx3 (green) antibodies were used to report the transgene expression; anti-activated caspase3 antibody was used to detect apoptotic cells (red). Gli3R was used as positive control of apoptosis. [file Image3.JPEG]
